# The *Arabidopsis thaliana* nucleotide sugar transporter GONST2 is a functional homolog of GONST1

**DOI:** 10.1002/pld3.309

**Published:** 2021-03-19

**Authors:** Beibei Jing, Toshiki Ishikawa, Nicole Soltis, Noriko Inada, Yan Liang, Gosia Murawska, Lin Fang, Fekadu Andeberhan, Ramana Pidatala, Xiaolan Yu, Edward Baidoo, Maki Kawai‐Yamada, Dominique Loque, Daniel J. Kliebenstein, Paul Dupree, Jenny C. Mortimer

**Affiliations:** ^1^ Joint BioEnergy Institute Emeryville CA USA; ^2^ Environmental Genomics and Systems Biology Division Lawrence Berkeley National Laboratory Berkeley CA USA; ^3^ Graduate School of Science and Engineering Saitama University Japan; ^4^ Plant Sciences Department UC Davis Davis CA USA; ^5^ Graduate School of Biological Sciences NAIST Nara Japan; ^6^ Department of Biochemistry University of Cambridge Cambridge UK; ^7^ School of Agriculture, Food and Wine University of Adelaide Adelaide SA Australia; ^8^ Present address: Graduate School of Life and Environmental Sciences Osaka Prefecture University Osaka Japan; ^9^ Present address: Chemistry Department Basel Switzerland; ^10^ Present address: Guangdong Provincial Key Laboratory of Applied Botany South China Botanical Garden Chinese Academy of Sciences Guangzhou China

**Keywords:** *Arabidopsis thaliana*, *Botrytis cinerea*, cell wall, GIPC, *Golovinomyces orontii*, sphingolipid, transporter

## Abstract

Glycosylinositolphosphorylceramides (GIPCs) are the predominant lipid in the outer leaflet of the plasma membrane. Characterized GIPC glycosylation mutants have severe or lethal plant phenotypes. However, the function of the glycosylation is unclear. Previously, we characterized *Arabidopsis thaliana* GONST1 and showed that it was a nucleotide sugar transporter which provides GDP‐mannose for GIPC glycosylation. *gonst1* has a severe growth phenotype, as well as a constitutive defense response. Here, we characterize a mutant in GONST1’s closest homolog, GONST2. The *gonst2‐*1 allele has a minor change to GIPC headgroup glycosylation. Like other reported GIPC glycosylation mutants, *gonst1‐1gonst2‐1* has reduced cellulose, a cell wall polymer that is synthesized at the plasma membrane. The *gonst2‐1* allele has increased resistance to a biotrophic pathogen *Golovinomyces orontii* but not the necrotrophic pathogen *Botrytis cinerea*. Expression of GONST2 under the GONST1 promoter can rescue the gonst1 phenotype, indicating that GONST2 has a similar function to GONST1 in providing GDP‐D‐Man for GIPC mannosylation.

## 1 INTRODUCTION

The plant plasma membrane is an asymmetric lipid bilayer which acts as both a selective barrier and a point of contact between the interior and exterior of the cell. Glycosylinositolphosphorylceramides (GIPCs) are a glycosylated form of sphingolipid, that comprise an estimated 64% of plant sphingolipids and ~25% of the total lipids in the *Arabidopsis thaliana* (Arabidopsis) leaf (Bure et al. 2011; Cacas et al. 2013, 2016; Markham & Jaworski, 2007; Markham et al. 2006, 2013). GIPCs are found predominantly in the outer leaflet of the plasma membrane.

GIPCs are a highly diverse class of lipids, comprising a long chain base (LCB) linked via an amide group to a fatty acid (FA) to form a ceramide, and a polar glycan head group. The diversity results from variation in the length, degree and position of unsaturation and hydroxylation of the FA and LCB, as well the structure and identity of the glycan head group. The ceramide is synthesized in the endoplasmic reticulum (ER), where it is either glucosylated to produce glucosylceramides, or it is then trafficked to the Golgi for GIPC biosynthesis. The first GIPC‐specific step is the addition of an inositol phosphate to the ceramide via headgroup exchange with a phospholipid phosphatidylinositol by inositolphosphorylceramide (IPC) synthase (IPCS) (Wang et al. 2008). The IPC core is first glycosylated with a glucuronic acid (GlcA) by the Carbohydrate Active enZyme (CAZy) family 8 glycosyltransferase (GT8) INOSITOL PHOSPHORYLCERAMIDE GLUCURONOSYLTRANSFERASE (IPUT1) (Rennie et al. 2014). The GlcA‐IPC core is further glycosylated to form mature GIPCs (Bure et al. 2011; Cacas et al. 2016; Fang et al. 2016; Ishikawa et al. ,,^2016^, ^2018^; Markham & Jaworski, 2007; Tellier et al. 2014). In Arabidopsis vegetative tissues, the dominant GIPC carries a mannose (Man) on the GlcA‐IPC, which is added by GIPC MANNOSYL‐TRANSFERASE1 (GMT1), from CAZy family GT64 (Fang et al. 2016). However, other glycosylated forms of GIPCs have also been identified. For example, in Arabidopsis seeds and pollen, rice, and tobacco leaves, the major GIPC glycosylation is a GlcN(Ac) linked to the GlcA, which can then be extensively decorated (Bure et al. 2011; Cacas et al. 2016; Carter et al. 1958; Hsieh et al. 1978, 1981; Ishikawa et al. 2018; Kaul & Lester, 1975, 1978; Luttgeharm et al. 2015; Tellier et al. 2014).

Recent evidence supports a role for GIPC glycosylation in plant–microbe interactions. For example, a peptide (NLP) which determines pathogenicity in many plant pathogens, including oomycetes, was shown to bind to the GIPC headgroup (Lenarcic et al. 2017). The degree of GIPC glycosylation was important in determining the degree of NLP cytotoxicity (Lenarcic et al. 2017). In *Medicago*, the type of GIPC glycosylation is important for the successful formation of root‐microbial symbioses, both with nodulating bacteria and arbuscular mycorrhizal fungi (Moore et al. submitted), and in Arabidopsis, plants with mutated GIPC glycosylation display a constitutive hypersensitive response, including elevated salicylic acid (SA) and reactive oxygen species (ROS; Fang et al. 2016; Mortimer et al. 2013).

In addition to GIPC glycosylation, many other glycosylation reactions occur in the lumen of the Golgi, including the synthesis of polysaccharides, and glycoproteins. Nucleotide sugars are the universal sugar donors for these processes. In plants, the majority of nucleotide sugars are UDP‐linked, but the GDP‐linked sugars GDP‐D‐Mannose (GDP‐Man), GDP‐D‐Glucose (GDP‐Glc), GDP‐L‐Fucose (GDP‐Fuc), and GDP‐L‐Galactose (GDP‐Gal) are also critical (Bar‐Peled & O'Neill, 2011). Most nucleotide sugars required in the Golgi, including all of the GDP‐sugars, are synthesized in the cytosol and therefore need to be translocated into the Golgi lumen via nucleotide sugar transporters (NSTs). Many of the Arabidopsis NSTs have now been heterologously characterized (Bakker et al. 2005; Baldwin et al. 2001; Ebert et al. 2015; Handford et al. 2004; Mortimer et al. 2013; Niemann et al. 2015; Norambuena et al. 2002, 2005; Rautengarten et al. ,^2014^, ^2016^, ^2017^; Reyes et al. 2010; Rollwitz et al. 2006; Saez‐Aguayo et al. 2017), although in vivo functionality is described for far fewer. Arabidopsis NSTs belong to the NST/triose phosphate translocator (TPT) superfamily which has 51 members that are distributed in six clades (Rautengarten et al. 2014). From this superfamily, only four members, the GOLGI LOCALIZED NUCLEOTIDE SUGAR TRANSPORTER (GONST) subclade, are predicted to transport GDP‐sugars due to the presence of the conserved GX[L/V]NK motif (Baldwin et al. 2001; Gao et al. 2001; Handford et al. 2004).

**FIGURE 1:**
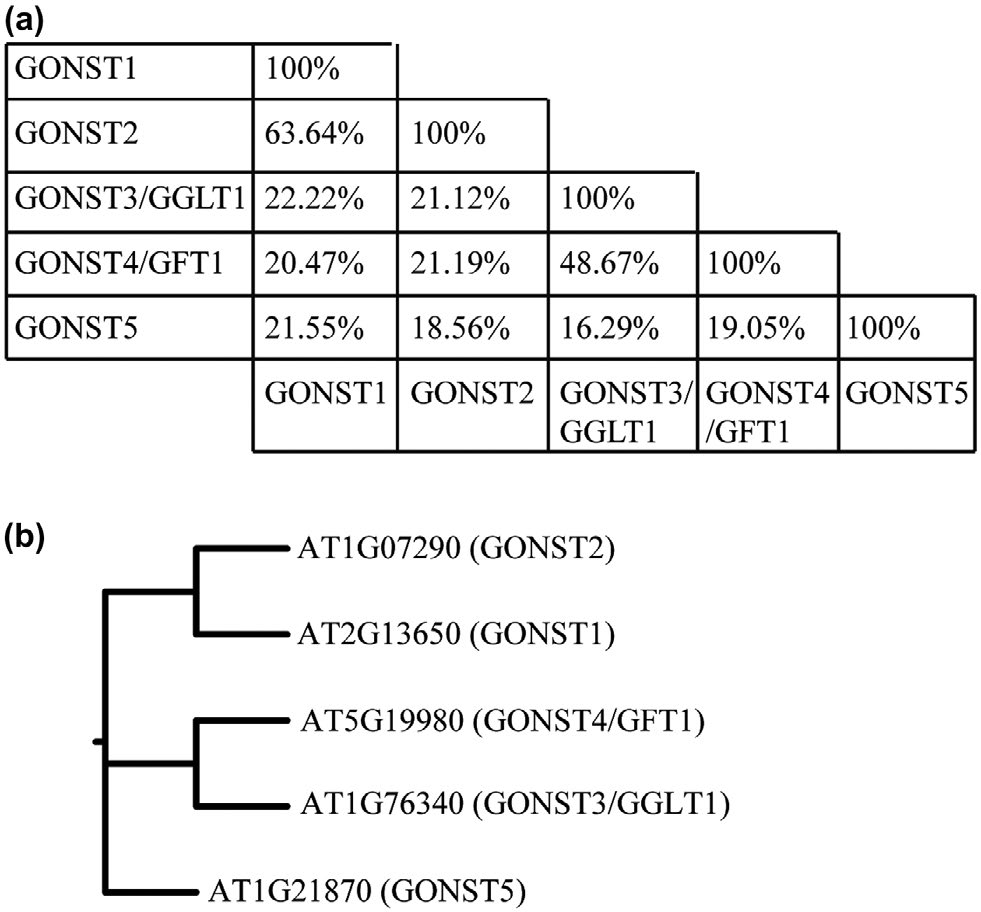
Phylogenetic characterization of Arabidopsis GONST family. Protein sequences of Arabidopsis GONST 1–5 were downloaded from TAIR (www.arabidopsis.org) and (a) aligned in Clustal Omega (standard parameters) in EMBL‐EBI to obtain the sequence identity. (b) The Phylip program set (v3.95) was used to build the tree, using standard parameters except where stated, as follows: seqboot (2000 replicates), proml (not rough analysis), consense, and drawgram. All bootstrap probabilities were 1.0 with 2000 replicates

The substrate for GMT1 is provided, at least in part, by GONST1, and indeed both *gonst1* and *gmt1* have very similar phenotypes (Fang et al. 2016; Mortimer et al. 2013). GONST1 was initially identified based on sequence similarity to *Saccharomyces cerevisiae* Vrg4p and *Leishmania donovani* LPG2 GDP‐Man transporters (Baldwin et al. 2001). GONST1 can complement the *Saccharomyces cerevisiae vrg4‐2* mutant and was the first biochemically characterized plant NST (Baldwin et al. 2001). GONST1 can transport all four plant GDP‐sugars in vitro (Mortimer et al. 2013). However, analysis of *gonst1* plants revealed a specific role in vivo as a GDP‐Man transporter which provides essential substrate for GIPC glycosylation (Figure 1) (Mortimer et al. 2013). GONST2 to GONST4 were identified as GONST1 homologues on the basis of their sequence similarity to GONST1 (Handford et al. 2004). GONST4 has now been characterized as the Golgi GDP‐Fuc transporter and has therefore been renamed GDP‐FUCOSE TRANSPORTER1 (GFT1) (Rautengarten et al. 2016). GONST3 has recently been shown to be responsible for GDP‐Gal transport and has been renamed GOLGI GDP‐L‐GALACTOSE TRANSPORTER1 (GGLT1) (Sechet et al. 2018). GONST2 was also able to complement *vrg4‐2* (Handford et al. 2004) and able to transport all four GDP‐linked sugars in vitro (Rautengarten et al. 2016), but its function *in planta*, as well as its specificity, remains unknown.

Here, we characterize the function of GONST2 *in planta* and demonstrate that even Arabidopsis plants with minor modifications to their GIPCs have increased resistance to a biotrophic pathogen (*Golovinomyces orontii*), but not to a necrotrophic pathogen (*Botrytis cinerea*). Expression of *GONST2* under the *GONST1* promoter can rescue the *gonst1* phenotype, indicating that GONST2 has a similar function to GONST1 in providing GDP‐Man for GIPC mannosylation.

## 2 MATERIALS AND METHODS

### 2.1 Materials

All constructs described in this publication are available upon request from the Joint BioEnergy Institute (JBEI)'s Inventory of Composable Elements (ICE) (registry.jbei.org). All chemicals are from Sigma Aldrich, unless otherwise noted. The GONST2 C‐terminal YFP construct pEarleygate101 GONST2 was a generous gift from Dr. Carsten Rautengarten, Lawrence Berkeley National Laboratory under the 35S promoter, and was described previously (Rautengarten et al. 2016).

### 2.2 Samples

All experiments were performed on at least three independently grown biological replicates unless otherwise stated.

### 2.3 Phylogenetics

Arabidopsis protein sequences were downloaded from TAIR (www.arabidopsis.org) and used in a BLASTp search (standard parameters) in NCBI. Sequences were aligned with Clustal Omega (www.ebi.org) (standard parameters). The Phylip program set (v3.95) was used to build the tree, using standard parameters except where stated, as follows: seqboot (2000 replicates), proml (not rough analysis), consense, and drawgram. All bootstrap probabilities were 1.0 with 2000 replicates.

### 2.4 Plant material and growth conditions

The T‐DNA line *gonst2‐1* (FLAG_406C01; ecotype Ws; insertion into AT1G07290) as well as *gonst1‐1* (FLAG_164D07; insertion into AT2G13650) were previously described in Mortimer et al. (2013). A second independent null *GONST2* T‐DNA insertion was not available, so two additional *gonst2* alleles were generated using CRISPR/Cas9 gene editing technology as described below. Arabidopsis seeds were surface sterilized and sown on solid medium containing 0.5x Murashige and Skoog salts including vitamins and 1% (w/v) sucrose. Following stratification (48 hr, 4°C, in the dark), plates were transferred to a growth room (22°C, 100–200 µmol/m^2^ s^–1^, 14 hr light/10 hr dark, 60% humidity). After 2–3 weeks, plants were transferred to soil or Magenta boxes under the same conditions. For *G. orontii* experiments, plants were grown under a 12 hr light/12 hr dark photoperiod for 4–5 weeks before inoculation. Liquid callus cultures were derived from Arabidopsis roots and maintained as described previously (Prime et al. 2000). *gonst2‐2* and *gonst2‐3* alleles are as described in Liang et al. (2019).

### 2.5 Subcellular localization

*Agrobacterium tumefaciens* (GV3101) transformed with either *35Spro*:*GONST2‐YFP* or the Golgi marker Man49‐GFP (Nelson et al. 2007) were co‐infiltrated into 4‐week‐old tobacco leaves. An additional *A. tumefaciens* strain carrying the p19 plasmid was also co‐infiltrated to stabilize the transgene expression. Forty‐eight hours after infiltration, the epidermal cells were removed from the tobacco leaves, fixed with formaldehyde and imaged using a Zeiss LSM 710 (Carl Zeiss, http://www.zeiss.com/) as previously outlined (Parsons et al. 2012). Image analysis and processing (scale bar, brightness, and contrast) were performed using IMAGEJ (Version 1.6r) (Schneider et al. 2012).

### 2.6 Histochemical detection of H_2_O_2_

Detection of H_2_O_2_ was by endogenous peroxidase‐dependent histochemical staining using 3,3‐diaminobenzidine (DAB) as described in Mortimer et al. (2013). Leaves of 15‐day‐old agar grown plants were submerged in 1 ml buffer (100 mM HEPES‐KOH, pH 6.8) or 1 mg/ml DAB in buffer. After 4 min of vacuum infiltration, leaves were incubated at 22°C under a light intensity of 100–200 µmol/m^2^ s^−1^ for 6 hr. Leaves were cleared for 30 min in 96% (v/v) ethanol solution at 70°C, and examined using a light microscope. Leaves were visually assessed as having either “no staining,” “light staining,” or “heavy staining.”

### 2.7 Quantitation of SA

For total SA determination, 500 mg leaves were frozen and ground in liquid nitrogen. The powder obtained was mixed with 1 ml 80% (v/v) methanol and incubated for 15 min at 70°C. This step was repeated four times. Pooled extracts were centrifuged and filtered through Amicon Ultra centrifugal filters (10,000 Da MW cutoff, EMD Millipore, Billerica, MA). The conjugated SA in the filtered extracts was dried and the hydrolyzed in 1 N HCl at 95°C for 3 hr. The mixture was subjected to three ethyl acetate partitioning steps. Ethyl acetate fractions were pooled, dried in vacuo, and resuspended in 50% (v/v) methanol. SA was quantified using HPLC‐electrospray ionization (ESI)‐time‐of‐flight (TOF) MS. Details of the running condition were described previously (Eudes et al. 2013).

### 2.8 *G. orontii* infection assay

*G. orontii* MGH was maintained on *pad4* leaves (Inada et al. 2016), and WT or *gonst2‐1* leaves were inoculated using a settling tower method, as previously described (Plotnikova et al. 1998). Five days after inoculation, leaves were collected ad cleared in 99% ethanol, stained with Trypan Blue (250 µg/ml Trypan Blue in 1:1:1 glycerol:lactic acid:water) for ~15 min at room temperature, destained (1:1:1 glycerol:lactic acid:water), and visualized under a light microscope. Three independent inoculation experiments were performed, and 12–30 leaves were used to count numbers of conidiophores per colony, for each genotype, in each experiment.

### 2.9 *B. cinerea* infection assay

*B. cinerea* inoculation followed a previously described protocol (Denby et al. 2004; Kliebenstein et al. 2005) using the following four strains: MEAP6G, 1.02.01, 1.03.01, and NobleRot. We grew seedlings in a randomized complete block design on soil (SunGro Horticulture, Agawam, MA) growth chambers in 20°C, short‐day (8h photoperiod) conditions. Spores were collected from mature *B. cinerea* cultures grown on canned peach plates and diluted to 10 spores/µL in filter‐sterilized 50% organic grape juice. At 7 weeks of age, we detached leaves from plants and arrayed them on 1% phytoagar by their order in the planting flats. We inoculated 4 uL spore solution droplets onto each leaf in a randomized complete block design, then incubated under 12 hr light/12 hr dark at room temperature for 96 hr. The spore solution was continuously agitated to ensure equal distribution of spores. Digital images were taken at 48, 72, and 96 hr post inoculation. Lesion area was measured using custom R scripts along with the EBImage and CRImage packages (Failmezger et al. 2012; Pau et al. 2010; RDevelopment CORE TEAM 2008).

### 2.10 GIPC analysis by TLC

Powdered, lyophilized liquid‐grown callus (200 mg) was added to 5 ml of the lower layer of isopropanol:hexane:water (55:20:25) and incubated at 50°C for 15 min. Following centrifugation (500 x *g*, 10 min), the supernatant was transferred to a fresh tube, and the pellet was re‐extracted with a further 5 ml of the lower layer of isopropanol:hexane:water (55:20:25). The supernatants were combined, dried under N2, and de‐esterified by incubation with 33% (v/v) methylamine in ethanol:water (7:3) at 50°C for 1 hr. After centrifugation (500 *g*, 10 min), the supernatant was retained, dried under N2, and incubated in 1 ml of chloroform:ethanol:ammonia:water (10:60:6:24) overnight at 21°C with agitation. Samples were subjected to weak anion exchange chromatography as described in Mortimer et al. (2013), and following elution from the cartridge were resuspended in chloroform:methanol:[4 M ammonium hydroxide in 1.8 M ammonium acetate] (9:7:2) and separated by thin layer chromatography (TLC) using high‐performance‐TLC Silica gel on glass plates (Merck) developed in the same buffer. GIPCs were visualized using primuline (Skipski, 1975).

### 2.11 GIPC analysis by LC/MS

Total lipid for sphingolipidomics was prepared from lyophilized tissues (5–10 mg dry weight) using a methanol/butanol‐based extraction coupled with weak alkaline hydrolysis and HCl treatment to remove glycerolipids and polysaccharides, respectively, according to the previous report (Ishikawa et al. 2018). Each sphingolipid species was quantified using LC‐MS/MS (LCMS‐8030, Shimadzu, Kyoto, Japan) with the MRM mode targeting glucosylceramides, free ceramides, and GIPCs with 0, 1, and 2 hexoses on GlcA‐IPCs. The contents of Hex‐GIPCs and ceramides were absolutely quantified by an internal standard‐based calculation method, and GlcA‐IPCs and Hex‐Hex‐GIPCs (for which we lack standards) were relatively quantified using the calculation factors as for Hex‐GIPCs as previously described (Fang et al. 2016; Ishikawa et al. 2016).

### 2.12 Cell wall monosaccharide analysis

AIR was prepared according to Mortimer et al. (2010) and 5 mg was hydrolyzed with fresh 2 M trifluoroacetic acid (TFA; 400 µl, 1 hr, 121°C). The supernatant was removed, and the pellet washed twice with water (400 µl). The supernatant and washings were combined, dried in vacuo, and analyzed by HPAEC‐PAD as previously described (Fang et al. 2016). The TFA‐insoluble pellet was subjected to Saeman hydrolysis. Briefly, following incubation in 72% (v/v) sulfuric acid (63 µl, 21°C, 1 hr), water was added to each sample to give a final sulfuric acid concentration of 1 M and incubated at 100°C for 3 hr. The samples were then neutralized with barium carbonate, to precipitate the sulfate ions, and the Glc content measured by HPAEC‐PAD as above.

### 2.13 Mannan structural analysis using PACE

PACE was performed according to Goubet et al. (2009) with slight modifications. Briefly, AIR (500 µg) was incubated with concentrated NH3 for 30 min at 21°C, and then dried in vacuo. Following resuspension in ammonium acetate buffer (0.1 M, 500 µl, pH 6.0), samples were incubated for 14 hr at 21°C with an excess of the mannanases CjMan5A and CjMan26A (a kind gift from Professor Harry Gilbert, University of Newcastle, UK). The released oligosaccharides were derivatized with 8‐aminonaphthalene‐1,3,6‐trisulfonic acid (Invitrogen) with 2‐picoline‐borane as the reducing agent, and separated by electrophoresis in large‐format polyacrylamide gels. Gels were visualized using a Syngene G:BOX gel doc system (Synoptics), equipped with long‐wave UV transilluminator bulbs and appropriate filters.

### 2.14 Promoter swap

The *GONST1* promoter (1.3 kb upstream of the start codon) and *GONST2* promoter (1.0 kb upstream of the start codon) were amplified by PCR from Col‐0 genomic DNA, and cloned into the binary vector pCAMBIA1305 to obtain pCAMBIA1305 *GONST1pro* and pCAMBIA1305 *GONST2pro*. Full‐length cDNA of *GONST2* were amplified by PCR and cloned into pCAMBIA1305 *GONST1pro* and pCAMBIA1305 *GONST2pro* to obtain pCAMBIA1305 *GONST1pro:GONST2* (Fusion 1) and pCAMBIA1305 *GONST2pro:GONST2* (Fusion 2). Constructs were transformed into A*grobacterium tumefasciens strain* GV3101 and used to transform *gonst1‐1* with the floral dip method. T3 plants, which were confirmed to be homozygous for the *gonst1‐1* T‐DNA insertion (Mortimer et al. 2013), were analyzed.

## 3 RESULTS

### 3.1 GONST2 is a close homolog of GONST1

Arabidopsis nucleotide‐sugar transporters containing a conserved GDP‐binding motif (GX[L/V]NK) were first identified by Baldwin et al. (2001) and named GONST, and consist of a clade of four proteins (GONST1‐2, GONST3/GGLT1, GONST4/GFT1) (Handford et al. 2004) (Figure 1). A fifth transporter (GONST5) is found in a distinct clade from GONST1‐4 and lacks the GXLNK (Handford et al. 2004; Rautengarten et al. 2014). GONST2 shares 61% identity with GONST1 at the amino acid level, as compared to only 19% with GFT1 (Figure 1), and is expressed at a low level in most tissues (Figure S1). We also confirmed that GONST2 is localized to the Golgi, as previously reported (Rautengarten et al. 2016) (Figure S2).

### 3.2 Use of CRISPR to generate new *gonst2* alleles

Previously, we isolated and partially characterized a homozygous *gonst2‐1* allele (Ws ecotype) which lacked detectable *GONST2* transcript by RT‐PCR but did not have a visible phenotype (Mortimer et al. 2013)). Since no further T‐DNA lines were available, we used CRISPR/Cas9 gene editing to create two further *gonst2* alleles, *gonst2‐2* and *gonst2‐3*, in the Col‐0 ecotype, as described in Liang et al. (2019) (Figure S3). As was the case for *gonst2‐1*, *gonst2‐2,* and *gonst2‐3* did not show a visible phenotype compared to WT.

### 3.3 Loss of GONST2 enhances the *gonst1* constitutive hypersensitive response

**FIGURE 2:**
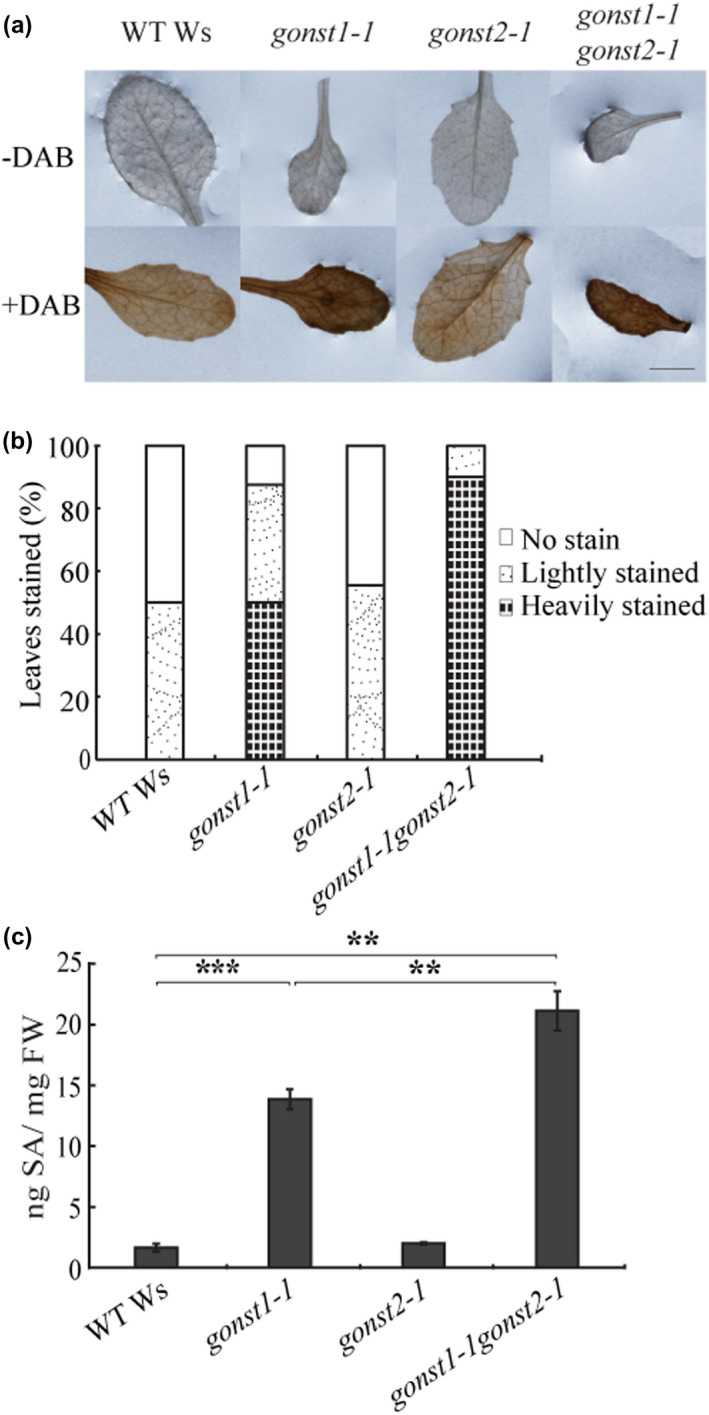
Loss of GONST2 enhances H_2_O_2_ and SA accumulation in the leaves of *gonst1* plants. (a) Intracellular H_2_O_2_ production was measured in 15‐day‐old leaves using DAB staining, and (b) the leaves were scored for staining intensity. (c) Total SA in 12‐day‐old leaves was quantitated by HPLC. All data are mean + *SD* of 3 independently grown biological replicates; Student's *t*‐test. Asterisk indicates a significant difference between the two indicated genotypes. ** *p* <.01, *** *p* <.001

*gonst1‐1gonst2‐1* has a more severe growth phenotype, as compared to *gonst1* alone (Figure S4) (Mortimer et al. 2013). *gonst1* has biochemical phenotypes consistent with the constitutive activation of plant defense responses, including elevated salicylic acid (SA) and reactive oxygen species (ROS) (Mortimer et al. 2013). To test whether loss of GONST2 also resulted in the constitutive activation of plant defense responses, we measured in situ H_2_O_2_ production using 2‐aminobenzidine +staining (DAB, as a proxy for ROS) and SA in *gonst2‐1*. *gonst2‐1* did not show a significant change in either SA or H_2_O_2_ production compared to WT (Figure 2). However, the *gonst1‐1gonst2‐1* double mutant showed increased frequency of heavier DAB staining and significantly higher salicylic acid as compared to WT and *gonst1‐1* alone (Figure 2).

### 3.4 *gonst2‐1* has increased resistance to a biotrophic pathogen but not to a necrotrophic pathogen

**FIGURE 3:**
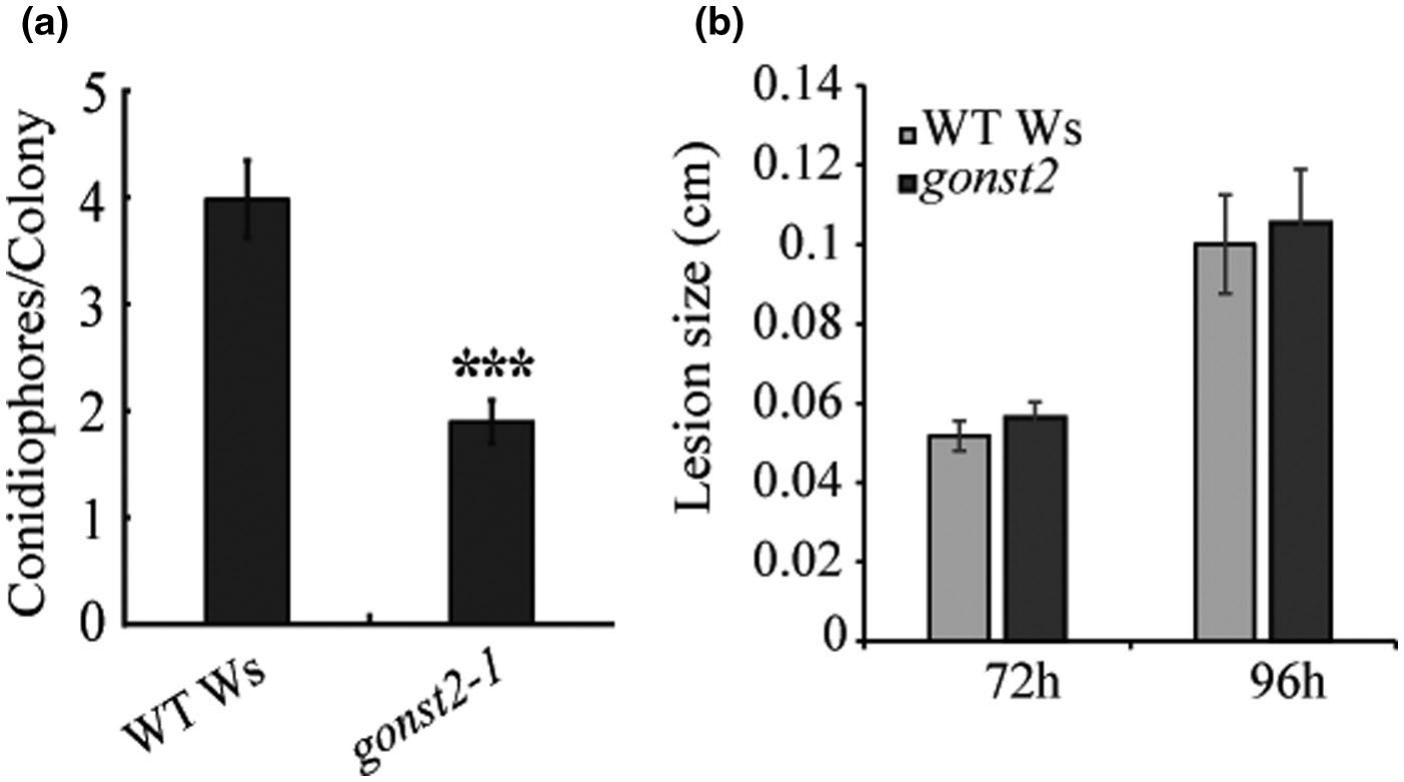
Susceptibility of *gonst2‐1* plants to biotrophic and necrotrophic pathogens. (a) 5 days after inoculation with the biotroph *G. orontii*, leaves were harvested, stained with trypan blue and conidiophores per colony counted. The data represent the mean of 12–30 leaves per genotype per experiment, scored in three independent experiments, ±*SD* (Student's *t*‐test, * *p* <.05, *** *p* <.001). (b) 72 or 96 hr after inoculation with four phenotypically diverse *B. cinerea* isolates (1.02, 1.03, MEAP6G, and NobleRot), lesion size was measured. The data represent the mean of 105–166 leaves per plant genotype per experiment, ±SE. No significant difference was detected between WT and *gonst2‐1* (*F*‐test; Table 1)

Plant pathogens can be divided into two major groups depending on their lifestyle strategies: necrotrophy and biotrophy. Necrotrophic pathogens kill host cells and extract nutrition from the dead host, while biotrophic pathogens colonize living cells and obtain nutrition from living hosts (Hammond‐Kosack & Jones, 1997). Classically, SA signaling triggers resistance against biotrophic pathogens, whereas a combination of jasmonic acid (JA) and ethylene (ET) signaling activates resistance against necrotrophic pathogens and these two pathways are mostly antagonistic (Robert‐Seilaniantz et al. 2011). We wanted to test whether *gonst1* or *gonst2* plants show increased pathogen resistance, and whether this was generic or specific to biotrophic pathogens. However, *gonst1‐1* and *gonst1‐1gonst2‐1* rosette leaves are not suitable for pathogen assays, as they are fully senesced by ~ 20 days under normal conditions. Therefore, we tested *gonst2‐1*, despite the lack of a detectably significant increase in SA or ROS (Figure 2), since its rosette leaves are healthy. Indeed, *gonst2‐1* showed a significant increase in resistance to the biotrophic pathogen *G. orontii* MGH (an Arabidopsis‐adapted powdery mildew) (Figure 3), as measured by conidiophores/colony. This was in contrast to pathoassays with the necrotrophic pathogen *Botrytis cinerea*. Four different isolates were tested, but there was no significant difference in susceptibility between WT and *gonst2‐1* (Figure 3; Table 1).

**TABLE 1:** ANOVA results for the various factors in the *Botrytis cinerea* infection experiment on WT and *gonst2* plants at 72 hr post inoculation

Sources of variation	*df*	SS	*p*
Botrytis isolates	3	1.623	<.001
WT versus. *gonst2*	1	0.00963	.3784
Time	1	0.04897	.1171
Isolate x plant genotype	3	0.2676	.8487

Random effects	*df*	LRT	*p*
Tray/flat	1	1.3844	.2394
Tray	1	9.1055	.002548

Abbreviations: df, degrees of freedoms; *p*, estimated *p*‐value; SS, Type III Sums‐of‐Squares.

### 3.5 *gonst2‐1* and *gonst1‐1gonst2‐1* have altered GIPC glycosylation

**FIGURE 4:**
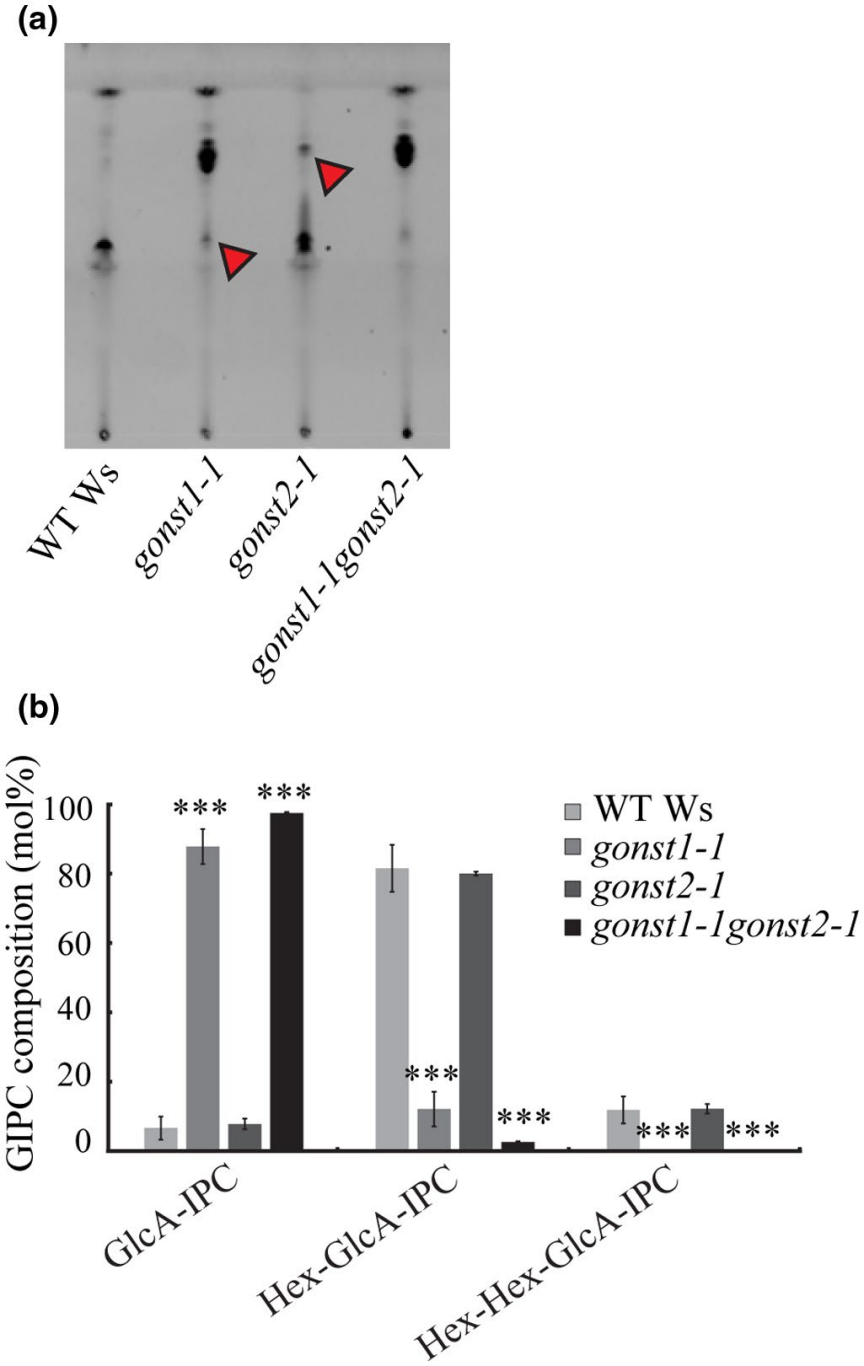
Glycan headgroup composition of *gonst* GIPCs (a) TLC of a GIPC‐enriched membrane fraction which has been stained with primuline. Bands discussed in the text are marked with a red arrow head. (b) An enriched GIPC fraction was analyzed by LC‐MS/MS MRM. The data here are collapsed to describe only the number of hexoses on the GIPC headgroup. All data are mean ± *SD* of three independently grown replicates of liquid grown cell culture; asterisk indicates significant difference from the wild type (Student's *t*‐test, *** *p* <.001). The full dataset is shown in Figure S5 and Dataset S1

Previously, we had shown that *gonst1* has reduced GIPC mannosylation (Mortimer et al. 2013). To explore GIPC glycosylation in *gonst2*, as well as *gonst1gonst2*, we developed a simple thin layer chromatography (TLC) method which separates GIPCs primarily due to the nature and the degree of glycosylation. Due to the small stature and tissue death in *gonst1* and *gonst1gonst2*, it was not possible to isolate GIPCs from whole plants. Therefore, we generated root‐derived callus from all of the genotypes, isolated an enriched GIPC fraction, and performed TLC. The plates were stained with primuline and visualized under UV light (Figure 4a). *gonst1‐1* was used as a control, and it shows a large shift in the mobility of the GIPCs, as compared to the Ws WT due to the loss of mannosylation as previously reported (Mortimer et al. 2013). A small fraction of the lower pool remains (marked with an arrow head). *gonst2‐1* has a profile similar to WT with a small upper fraction of GIPCs (marked with an arrow head), and *gonst1‐1gonst2‐1* had essentially all GIPCs in the faster moving upper fraction.

To explore this further, we then used LC‐MS/MS multiple reaction monitoring (MRM) to perform sphingolipidomics. No overall significant difference was detected in the glucosylceramides, hydroxyceramides or ceramides (Figure S5, Dataset S1). Plant GIPCs are enormously complex, due to the possible variations in FA, LCB, and glycan structure. Since no changes in the total amount of GIPCs nor the ceramide composition were detected (Figure S5). Following the nomenclature described in Fang et al. (2016) (Figure S6), the data have been aggregated to show the relative amount of GIPCs containing either 0, 1, or 2 hexoses terminal to GlcA‐IPC (Figure 4b). While *gonst2‐1* did not show a significantly different GIPC headgroup profile from the WT, there was significantly less Man‐GlcA‐GIPCs in *gonst1‐1gonst2‐1* than *gonst1‐1* (*t*‐test, *p* =.04, Figure 4b, Dataset S1).

### 3.6 *gonst2‐1* and *gonst1‐1gonst2‐1* Golgi‐synthesized cell wall polysaccharides are unaffected

Since GONST2 is a Golgi‐localized nucleotide sugar transporter, we next tested whether the loss of GONST2 could impact other glycosylation processes in the Golgi, in addition to GIPCs. The majority of cell wall polysaccharide biosynthesis, with the exception of cellulose and callose, occurs in the Golgi, so we investigated the monosaccharide composition of the non‐cellulosic polysaccharides of callus, leaves, and stems by hydrolyzing an alcohol insoluble residue (AIR) cell wall preparation with trifluoroacetic acid (TFA) (Figure S7). No significant difference was detected between *gonst2‐1*, *gonst1‐1gonst2‐1,* and the WT.

### 3.7 *gonst2‐1* and *gonst1‐1gonst2‐1* glucomannan structure and quantity are unchanged

**FIGURE 5:**
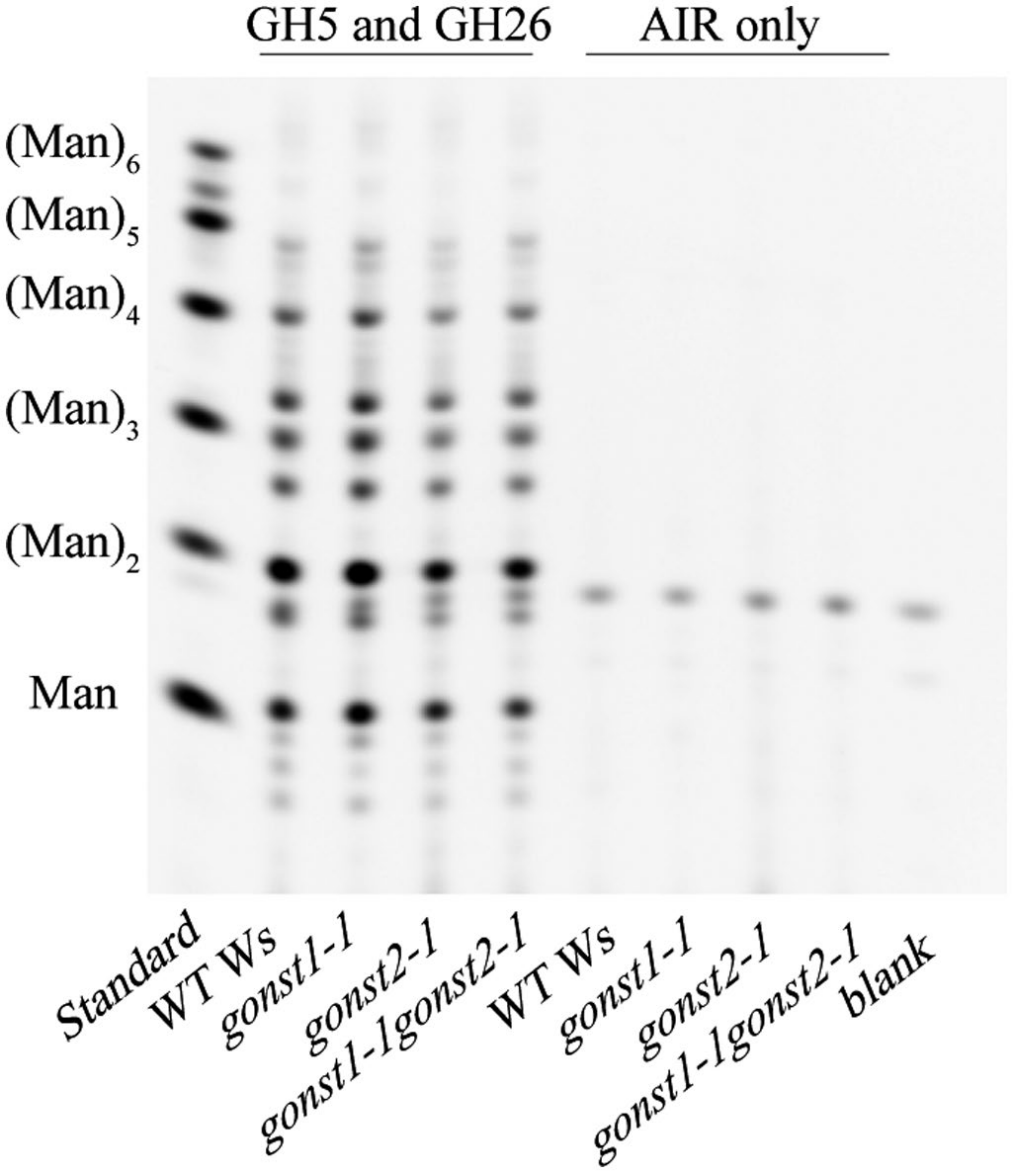
PACE fingerprint of mannan in stem cell walls of WT, *gonst1*, *gonst2,* and *gonst1gonst2*. Oligosaccharides released from AIR by mannanase digestion were derivatized with 8‐aminonapthalene‐1,3,6‐trisulfonic acid (ANTS) and visualized by PACE. (Man)_1‐6_ oligosaccharides were used as a standard. A representative gel from multiple experiments is shown

Glucomannan is a Golgi‐synthesized cell wall polysaccharide composed of β(1,4)‐Man and –Glc, which is synthesized by the CSLA family of GTs. CSLA9 (the dominant mannan synthase in Arabidopsis vegetative tissue) requires GDP‐Man and GDP‐Glc for glucomannan synthesis (Dhugga et al. 2004; Goubet et al. 2009; Liepman et al. 2005), and it has been proposed that it has a luminal active site (Davis et al. 2010). However, no NST responsible for providing these substrates to the Golgi lumen has yet been identified. Loss of GONST1 does not affect glucomannan biosynthesis (Mortimer et al. 2013), GFT1 is a GDP‐Fuc transporter (Rautengarten et al. 2016), and GGLT1 is a GDP‐Gal transporter (Sechet et al. 2018). Since glucomannan is a relatively minor component of the cell wall (Handford et al. 2003), the monosaccharide analysis may not reveal alterations to its quantity or the Glc:Man ratio of the glucomannan backbone. Therefore, we used Polysaccharide Analysis by Carbohydrate gel Electrophoresis (PACE) to investigate glucomannan quantity and structure (Handford et al. 2003). No difference was seen either in the type or quantity of the oligosaccharides released by hydrolysis of *gonst2‐1, gonst1‐1gonst2‐1,* and WT AIR by mannanases (Figure 5). Therefore, this suggests that GONST1 and GONST2 are not providing substrate in the Golgi lumen for mannan biosynthesis.

### 3.8 *gonst1‐1gonst2‐1* has less cellulose

**FIGURE 6:**
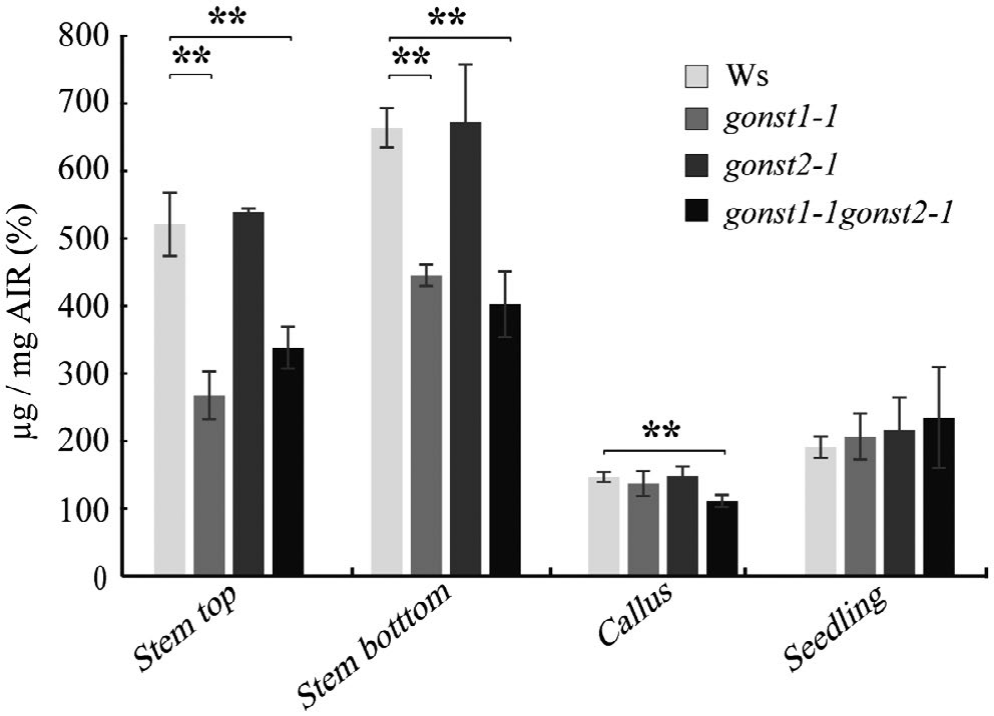
*gonst1gonst2* has reduced crystalline cellulose. Crystalline cellulose content was determined as the glucose content released by sulfuric acid treatment of the TFA‐insoluble fraction of AIR. All data are mean ± *SD* of three biological replicates. Asterisk indicates significant difference from the wild type (Student's *t*‐test, ** *p* <.01)

Previously, we showed that a mutant in GIPC mannosylation (GMT1) has reduced cellulose (Fang et al. 2016). To test whether this phenotype is common to plants with altered GIPC mannosylation, we hydrolyzed the TFA‐insoluble AIR fraction with sulfuric acid to release glucose derived from cellulose (Figure 6). *gonst1‐1* had a significant reduction in upper and lower stem cellulose content as compared to WT, whereas callus and seedling were unaffected. *gonst2‐1* did not show a significant difference in any tissue type analyzed compared to WT. However, *gonst1‐1gonst2‐1* mutants showed a significant decrease in callus cellulose content (a tissue rich in primary cell wall), compared to the WT or single mutants, but not in other tissue types. These data are consistent with a specific role for GIPC mannosylation in determining cell wall cellulose content.

### 3.9 Expression of *GONST1pro:GONST2* in *gonst1‐1* rescues growth and GIPC glycosylation

**FIGURE 7:**
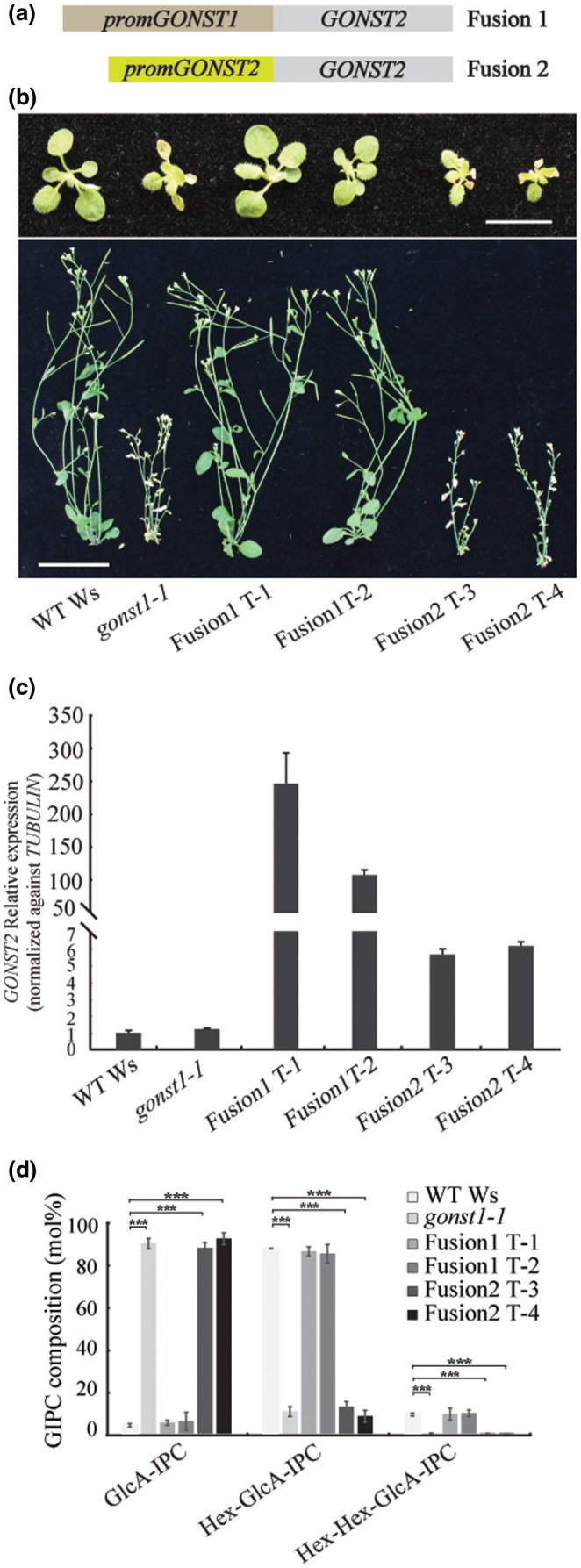
Expression of *GONST2* under the *GONST1* promoter in the *gonst1* background restores the dwarfed phenotype of *gonst1*. (a) Schematic of the two introduced constructs. (b) Top row: 15‐day‐old, agar grown WT, *gonst1‐1*, Fusion1 T‐1, Fusion1 T‐2, Fusion2 T‐3, and Fusion2 T‐4 seedlings. Scale bar = 1 cm. Bottom row: 6‐week‐old WT, *gonst1‐1*, Fusion1 T‐1, Fusion1 T‐2, Fusion2 T‐3, and Fusion2 T‐4. Plants were first grown on agar for 10 days, and then transplanted onto soil. Bar = 3 cm. (c) Gene expression analysis of *GONST2* relative to WT Ws and normalized against TUBULIN using Q‐PCR. Values represent average of three biological replicates ±*SD*. (d) An enriched GIPC fraction was analyzed by LC‐MS/MS MRM. The data here are collapsed to describe only the number of hexoses on the GIPC headgroup. All data are mean ± *SD* of three independently grown replicates of liquid grown cell culture. Asterisk indicates significant difference from the wild type (Student's *t*‐test, * *p* <.05, *** *p* <.001)

To test whether the phenotypic differences observed between *gonst1* and *gonst2* are due to differences in the protein function, or whether they are due to differences in expression level, we expressed the *GONST2* coding sequence (CDS) driven by either the *GONST1* promoter (*GONST1pro:GONST2*) or the *GONST2* promoter (*GONST2pro:GONST2*) in the *gonst1‐1* background. Multiple independently transformed lines were selected for analysis. Analysis of T3 segregants revealed that some of the homozygous *gonst1‐1* plants had a restored growth phenotype (Figure 7). *GONST2* expression was analyzed by real‐time RT‐PCR (Figure 7). The suppression of the *gonst1‐1* growth phenotype was only apparent in those lines in which GONST2 expression was driven by the GONST1 promoter (Figure 7). The rescue of the growth phenotype was reflected in the biochemical characterization of GIPC headgroup composition (Figure 7). This result supports the view that GONST2 has the same function as GONST1, and that the function is cell type specific and/or dose dependent.

## 4 DISCUSSION

The aim of this research was to characterize the role of the final *bona fide* member of the GONST clade of four GDP‐sugar transporters, GONST2. Our data support the conclusion that, while in vitro it has been reported to transport all GDP‐linked sugars (Rautengarten et al. 2016), *in planta* it has a specific role in providing GDP‐Man for GIPC glycosylation. We also show that GONST2 is a functional homolog of GONST1. Since *gonst2* does not display the severe growth defects of *gonst1*, we were able to perform pathoassays to investigate the previously reported constitutive defense response of *gonst1* (Mortimer et al. 2013), and show that *gonst2‐1* has increased resistance to the biotrophic pathogen *G. orontii* but not the necrotrophic pathogen *B. cinerea*. Note that these assays were only performed on a single allele of *gonst2*, and so remain to be confirmed using the additional gonst2 alleles or complementation lines.

Resistance to biotrophic pathogens, such as the powdery mildew‐causing *G. orontii,* is regulated by SA signaling (Wildermuth et al. 2001). Activation of SA signaling is often correlated with accumulation of reactive oxygen species including H_2_O_2_ (Herrera‐Vasquez et al. 2015). Resistance to necrotrophic pathogens such as *B. cinerea* requires JA/ET signaling, which mostly function antagonistically with SA (Robert‐Seilaniantz et al. 2011). We found that the *gonst1‐1gonst2‐1* double mutant contains significantly increased SA and enhanced H_2_O_2_ accumulation. While uninfected *gonst2‐1* did not show an increased SA level, it is possible that the SA level is enhanced in *gonst2‐1* after *G. orontii* infection, contributing to the enhanced resistance to *G. orontii*. It has been reported that altered ceramide profiles are associated with altered phytohormone levels, and thus with an altered response to pathogens (Magnin‐Robert et al. 2015). In this case, ceramide functions as a signaling component. While *gonst2* does not show a significant change to the ceramide pool, changes to the GIPC glycosylation may be enough to affect SA signaling and thus the response to *G. orontii*. Alternatively, a defect in membrane trafficking in *gonst2‐1* may negatively impact *G. orntii* infection. *G. orontii* forms a specialized infection hypha called the haustorium in the host apoplast to establish infection. The haustorium is surrounded by host‐derived membrane called the extrahaustorial membrane, which has modified endosomal characteristics (Inada, Betsuyaku, et al., 2016). It has been shown that GIPCs are important for secretory sorting of proteins (Markham et al. 2011; Wattelet‐Boyer et al. 2016), and therefore, it may be that changes to a minor class of GPIC are enough to disrupt these processes, thereby negatively affecting *G. orontii* infection. GIPC glycan engineering therefore offers a promising approach for developing plants with increased disease resistance.

Cellulose content is decreased in *gonst1‐1* and *gonst1‐1gonst2‐1* plants. Cellulose is synthesized at the plasma membrane by rosettes of CESA proteins which move through the plane of the plasma membrane (McFarlane et al. 2014). The rosettes are assembled in the Golgi and are delivered to the plasma membrane via the secretory system (Wightman & Turner, 2010). The reduced cellulose phenotype was also reported for *gmt1*, which has the same biochemical GIPC phenotype as *gonst1gonst2* (Fang et al. 2016). The reasons for this decrease are not clear. It is possible that the altered GIPC glycosylation affects trafficking of the rosettes to the plasma membrane, or alternatively, the change to plasma membrane composition affects CESA function. CESA proteins are S‐acylated, and it has been suggested that this decoration may either localize proteins to lipid microdomains (which are rich in GIPCs) or even facilitate their formation (Konrad & Ott, 2015; Kumar et al. 2016). COBRA and COBRA‐like proteins which are also essential for normal cellulose biosynthesis are glycosylinositolphosphatidylinositol (GPI) anchored (Roudier et al. 2005). GPI anchored proteins are targeted to the outer leaflet of the plasma membrane, and to lipid microdomains (Borner et al. 2005). Therefore, correct GIPC glycosylation may be necessary for either CESA activity or localization and retention of GPI‐anchored proteins in the plasma membrane. More recently, a role for GIPCs in modulating the salt‐dependent activation of a plasma membrane calcium channel (Jiang et al. 2019) and for plasmodesmata function (Yan et al. 2019) suggesting that GIPCs may have a broad role in regulating plasma membrane functionality.

It should be noted that alterations to cellulose content can affect susceptibility to some pathogens (Hernandez‐Blanco et al. 2007; Malinovsky et al. 2014). However, some difference has been observed between whether primary wall or secondary wall cellulose is impaired, and the type of immune response that is induced (Bacete et al. 2018). For example, CESA3 mutants (a primary cell wall CESA) are more resistant to powdery mildews (Cano‐Delgado et al. 2003; Ellis & Turner, 2001) and mutants in secondary cell wall CESAs and secondary cell wall deposition are more resistant to necrotrophs (Hernandez‐Blanco et al. 2007; Ramirez et al. 2011).

Mannan content is unchanged in the *gonst1gonst2* plants. It had been reported that CSLA9, unlike related GT2 proteins (CSLC4, CESAs), has a topology which results in a luminal active site (Davis et al. 2010). This would necessarily require a nucleotide transporter to provide GDP‐sugars for mannan biosynthesis. However, none of the predicted GDP‐sugar transporters seem to have this function *in planta* (Mortimer et al. 2013; Rautengarten et al. 2016; Sechet et al. 2018). This implies that either the mannan synthases do not require a nucleotide sugar transporter, or that the transporter does not have a canonical GDP‐binding motif.

Future work will be required to confirm these data, making use of the additional *gonst2* alleles now available (Liang et al. 2019). It will also be important to establish how GIPC glycosylation affects these assorted membrane‐based processes. For example, molecular dynamics could be applied to model the plant plasma membrane and understand how the GIPC glycan headgroup structure affects protein movement within the membrane. It will also be interesting to understand what drives the differences in functionality of the NSTs in in vitro assays versus *in planta* function. Both GONST1 and GONST2 can transport all GDP‐sugars when tested in liposome‐based assays (Mortimer et al. 2013; Rautengarten et al. 2016), but it is clear that they are highly specific in vivo. This could be mediated by substrate concentration, interaction with non‐catalytic proteins, or interactions with the GT that utilizes the substrate (in this case GMT1 (Fang et al. 2016)). The recent crystal structure of the yeast Vrg4 NST provided new insights into how NST function is regulated (Parker & Newstead, 2017). To our knowledge, no plant NSTs have yet been structurally characterized, but we expect that this information will be critical for understanding NST specificity.

## CONFLICTS OF INTEREST

No conflicts of interest to declare.

## AUTHOR CONTRIBUTIONS

JCM and BJ designed the research. BJ, JCM, FA, GM, LF, and RP carried out the research. TI performed sphingolidomics; NS and NI performed pathogen assays; EB analyzed salicylic acid quantities; YL generated CRISPR lines; XY analyzed mannan structure by PACE. JCM, BJ, TI, NS, NI, MKY, DL, and PD analyzed data.

## Supporting information

FigS1

FigS2

FigS3

FigS4

FigS5

FigS6

FigS7

TableS1

DatasetS1

DatasetS2

## References

[pld3309-bib-0001] Bacete, L. , Melida, H. , Miedes, E. , & Molina, A. (2018). Plant cell wall‐mediated immunity: Cell wall changes trigger disease resistance responses. The Plant Journal, 93, 614–636. 10.1111/tpj.13807 29266460

[pld3309-bib-0002] Bakker, H. , Routier, F. , Oelmann, S. , Jordi, W. , Lommen, A. , Gerardy‐Schahn, R. , Bosch D. . (2005). Molecular cloning of two Arabidopsis UDP‐galactose transporters by complementation of a deficient Chinese hamster ovary cell line. Glycobiology, 15, 193–201. 10.1093/glycob/cwh159 15456736

[pld3309-bib-0003] Baldwin, T. C. , Handford, M. G. , Yuseff, M. I. , Orellana, A. , & Dupree, P. (2001). Identification and characterization of GONST1, a Golgi‐localized GDP‐mannose transporter in Arabidopsis. The Plant Cell, 13, 2283–2295. 10.1105/tpc.010247 11595802 PMC139159

[pld3309-bib-0004] Bar‐Peled, M. , & O'Neill, M. A. (2011). Plant nucleotide sugar formation, interconversion, and salvage by sugar recycling. Annual Review of Plant Biology, 62, 127–155. 10.1146/annurev-arplant-042110-103918 21370975

[pld3309-bib-0005] Borner, G. H. H. , Sherrier, D. J. , Weimar, T. , Michaelson, L. V. , Hawkins, N. D. , MacAskill, A. , Napier, J. A. , Beale, M. H. , Lilley, K. S. , & Dupree, P. (2005). Analysis of detergent‐resistant membranes in Arabidopsis. Evidence for plasma membrane lipid rafts. Plant Physiology, 137, 104–116. 10.1104/pp.104.053041 15618420 PMC548842

[pld3309-bib-0006] Buré, C. , Cacas, J.‐L. , Wang, F. , Gaudin, K. , Domergue, F. , Mongrand, S. , & Schmitter, J.‐M. (2011). Fast screening of highly glycosylated plant sphingolipids by tandem mass spectrometry. Rapid Communications in Mass Spectrometry, 25, 3131–3145. 10.1002/rcm.5206 21953969

[pld3309-bib-0007] Cacas, J.‐L. , Buré, C. , Furt, F. , Maalouf, J.‐P. , Badoc, A. , Cluzet, S. , Schmitter, J.‐M. , Antajan, E. , & Mongrand, S. (2013). Biochemical survey of the polar head of plant glycosylinositolphosphoceramides unravels broad diversity. Phytochemistry, 96, 191–200. 10.1016/j.phytochem.2013.08.002 23993446

[pld3309-bib-0008] Cacas, J.‐L. , Buré, C. , Grosjean, K. , Gerbeau‐Pissot, P. , Lherminier, J. , Rombouts, Y. , Maes, E. , Bossard, C. , Gronnier, J. , Furt, F. , Fouillen, L. , Germain, V. , Bayer, E. , Cluzet, S. , Robert, F. , Schmitter, J.‐M. , Deleu, M. , Lins, L. , Simon‐Plas, F. , & Mongrand, S. (2016). Revisiting Plant Plasma Membrane Lipids in Tobacco: A Focus on Sphingolipids. Plant Physiology, 170, 367–384. 10.1104/pp.15.00564 26518342 PMC4704565

[pld3309-bib-0009] Cano‐Delgado, A. , Penfield, S. , Smith, C. , Catley, M. , & Bevan, M. (2003). Reduced cellulose synthesis invokes lignification and defense responses in Arabidopsis thaliana. Plant Journal, 34, 351–362. 10.1046/j.1365-313X.2003.01729.x 12713541

[pld3309-bib-0010] Carter, H. E. , Gigg, R. H. , Law, J. H. , Nakayama, T. , & Weber, E. (1958). Biochemistry of the sphingolipides. XI. Structure of Phytoglycolipide. Journal of Biological Chemistry, 233, 1309–1314. 10.1016/S0021-9258(18)49332-5 13610833

[pld3309-bib-0011] Davis, J. , Brandizzi, F. , Liepman, A. H. , & Keegstra, K. (2010). Arabidopsis mannan synthase CSLA9 and glucan synthase CSLC4 have opposite orientations in the Golgi membrane. The Plant Journal, 64, 1028–1037. 10.1111/j.1365-313X.2010.04392.x 21143682

[pld3309-bib-0012] Denby, K. J. , Kumar, P. , & Kliebenstein, D. J. (2004). Identification of Botrytis cinerea susceptibility loci in Arabidopsis thaliana. The Plant Journal, 38, 473–486 15086796 10.1111/j.0960-7412.2004.02059.x

[pld3309-bib-0013] Dhugga, K. S. , Barreiro, R. , Whitten, B. , Stecca, K. , Hazebroek, J. , Randhawa, G. S. , Dolan, M. , Kinney, A. J. , Tomes, D. , Nichols, S. , & Anderson, P. (2004). Guar seed beta‐mannan synthase is a member of the cellulose synthase super gene family. Science, 303, 363–366. 14726589 10.1126/science.1090908

[pld3309-bib-0014] Ebert, B. , Rautengarten, C. , Guo, X. , Xiong, G. , Stonebloom, S. , Smith‐Moritz, A. M. , Herter, T. , Chan, L. J. G. , Adams, P. D. , Petzold, C. J. , Pauly, M. , Willats, W. G. T. , Heazlewood, J. L. , & Scheller, H. V. (2015). Identification and characterization of a golgi‐localized UDP‐Xylose transporter family from arabidopsis. The Plant Cell, 27, 1218–1227. 10.1105/tpc.114.133827 25804536 PMC4558686

[pld3309-bib-0015] Ellis, C. , & Turner, J. G. (2001). The Arabidopsis mutant cev1 has constitutively active jasmonate and ethylene signal pathways and enhanced resistance to pathogens. The Plant Cell, 13, 1025–1033. 11340179 10.1105/tpc.13.5.1025 PMC135553

[pld3309-bib-0016] Eudes, A. , Juminaga, D. , Baidoo, E. E. , Collins, F. W. , Keasling, J. D. , & Loque, D. (2013). Production of hydroxycinnamoyl anthranilates from glucose in *Escherichia coli* . Microbial Cell Factories, 12, 62.– 10.1186/1475-2859-12-62 23806124 PMC3716870

[pld3309-bib-0017] Failmezger, H. , Yuan, Y. , Rueda, O. , & Markowetz, F. (2012). CRImage: CRImage a package to classify cells and calculate tumour cellularity. R package version 1.28.0.

[pld3309-bib-0018] Fang, L. , Ishikawa, T. , Rennie, E. A. , Murawska, G. M. , Lao, J. , Yan, J. , Tsai, A.‐L. , Baidoo, E. E. K. , Xu, J. , Keasling, J. D. , Demura, T. , Kawai‐Yamada, M. , Scheller, H. V. , & Mortimer, J. C. (2016). Loss of inositol phosphorylceramide sphingolipid mannosylation induces plant immune responses and reduces cellulose content in arabidopsis. The Plant Cell, 28, 2991–3004. 10.1105/tpc.16.00186 27895225 PMC5240734

[pld3309-bib-0019] Gao, X. D. , Nishikawa, A. , & Dean, N. (2001). Identification of a conserved motif in the yeast golgi GDP‐mannose transporter required for binding to nucleotide sugar. Journal of Biological Chemistry, 276, 4424–4432. 10.1074/jbc.M009114200 11067855

[pld3309-bib-0020] Goubet, F. , Barton, C. J. , Mortimer, J. C. , Yu, X. , Zhang, Z. , Miles, G. P. , Richens, J. , Liepman, A. H. , Seffen, K. , & Dupree, P. (2009). Cell wall glucomannan in Arabidopsis is synthesised by CSLA glycosyltransferases, and influences the progression of embryogenesis. The Plant Journal, 60, 527–538. 10.1111/j.1365-313X.2009.03977.x 19619156

[pld3309-bib-0021] Hammond‐Kosack, K. E. , & Jones, J. D. (1997). Plant disease resistance genes. Annual Review of Plant Physiology and Plant Molecular Biology, 48, 575–607. 10.1146/annurev.arplant.48.1.575 15012275

[pld3309-bib-0022] Handford, M. G. , Baldwin, T. C. , Goubet, F. , Prime, T. A. , Miles, J. , Yu, X. , & Dupree, P. (2003). Localisation and characterisation of cell wall mannan polysaccharides in Arabidopsis thaliana. Planta, 218, 27–36. 10.1007/s00425-003-1073-9 12844268

[pld3309-bib-0023] Handford, M. G. , Sicilia, F. , Brandizzi, F. , Chung, J. H. , & Dupree, P. (2004). *Arabidopsis thaliana* expresses multiple Golgi‐localised nucleotide‐sugar transporters related to GONST1. Molecular Genetics and Genomics, 272, 397–410. 10.1007/s00438-004-1071-z 15480787

[pld3309-bib-0024] Hernandez‐Blanco, C. , Feng, D. X. , Hu, J. , Sanchez‐Vallet, A. , Deslandes, L. , Llorente, F. , Berrocal‐Lobo, M. , Keller, H. , Barlet, X. , Sánchez‐Rodríguez, C. , & Anderson, L. K. (2007). Impairment of cellulose synthases required for Arabidopsis secondary cell wall formation enhances disease resistance. The Plant Cell, 19, 890–903. 17351116 10.1105/tpc.106.048058 PMC1867366

[pld3309-bib-0025] Herrera‐Vasquez, A. , Salinas, P. , & Holuigue, L. (2015). Salicylic acid and reactive oxygen species interplay in the transcriptional control of defense genes expression. Frontiers in Plant Science, 6, 171., 10.3389/fpls.2015.00171 25852720 PMC4365548

[pld3309-bib-0026] Hsieh, T. C. , Kaul, K. , Laine, R. A. , & Lester, R. L. (1978). Structure of a major glycophosphoceramide from tobacco leaves, PSL‐I: 2‐deoxy‐2‐acetamido‐D‐glucopyranosyl(alpha1 leads to 4)‐D‐glucuronopyranosyl(alpha1 leads to 2)myoinositol‐1‐O‐phosphoceramide. Biochemistry, 17, 3575–3581. 210797 10.1021/bi00610a024

[pld3309-bib-0027] Hsieh, T. C. , Lester, R. L. , & Laine, R. A. (1981). Glycophosphoceramides from plants. Purification and characterization of a novel tetrasaccharide derived from tobacco leaf glycolipids. Journal of Biological Chemistry, 256, 7747–7755. 10.1016/S0021-9258(18)43340-6 7263625

[pld3309-bib-0028] Inada, N. , Betsuyaku, S. , Shimada, T. L. , Ebine, K. , Ito, E. , Kutsuna, N. , Hasezawa, S. , Takano, Y. , Fukuda, H. , Nakano, A. , & Ueda, T. (2016). Modulation of plant RAB GTPase‐mediated membrane trafficking pathway at the interface between plants and obligate biotrophic pathogens. Plant & Cell Physiology, 57, 1854–1864. 10.1093/pcp/pcw107 27318282

[pld3309-bib-0029] Inada, N. , Higaki, T. , & Hasezawa, S. (2016). Nuclear function of subclass I actin‐depolymerizing factor contributes to susceptibility in arabidopsis to an adapted powdery mildew fungus. Plant Physiology, 170, 1420–1434. 10.1104/pp.15.01265 26747284 PMC4775110

[pld3309-bib-0030] Ishikawa, T. , Fang, L. , Rennie, E. A. , Sechet, J. , Yan, J. , Jing, B. , Moore, W. , Cahoon, E. B. , Scheller, H. V. , Kawai‐Yamada, M. , & Mortimer, J. C. (2018). GLUCOSAMINE INOSITOLPHOSPHORYLCERAMIDE TRANSFERASE1 (GINT1) is a GlcNAc‐containing glycosylinositol phosphorylceramide glycosyltransferase. Plant Physiology, 177(3), 938–952. 10.1104/pp.18.00396 29760197 PMC6053017

[pld3309-bib-0031] Ishikawa, T. , Ito, Y. , & Kawai‐Yamada, M. (2016). Molecular characterization and targeted quantitative profiling of the sphingolipidome in rice. The Plant Journal, 88, 681–693. 10.1111/tpj.13281 27454201

[pld3309-bib-0032] Jiang, Z. , Zhou, X. , Tao, M. , Yuan, F. , Liu, L. , Wu, F. , Wu, X. , Xiang, Y. , Niu, Y. , Liu, F. , Li, C. , Ye, R. , Byeon, B. , Xue, Y. , Zhao, H. , Wang, H.‐N. , Crawford, B. M. , Johnson, D. M. , Hu, C. , … Pei, Z.‐M. (2019). Plant cell‐surface GIPC sphingolipids sense salt to trigger Ca2+ influx. Nature, 572, 341–346. 10.1038/s41586-019-1449-z 31367039

[pld3309-bib-0033] Kaul, K. , & Lester, R. L. (1975). Characterization of inositol‐containing phosphosphingolipids from tobacco leaves: isolation and identification of two novel, major lipids: N‐acetylglucosamidoglucuronidoinositol phosphorylceramide and glucosamidoglucuronidoinositol phosphorylceramide. Plant Physiology, 55, 120–129. 10.1104/pp.55.1.120 16659016 PMC541565

[pld3309-bib-0034] Kaul, K. , & Lester, R. L. (1978). Isolation of six novel phosphoinositol‐containing sphingolipids from tobacco leaves. Biochemistry, 17, 3569–3575. 10.1021/bi00610a023 687599

[pld3309-bib-0035] Kliebenstein, D. J. , Rowe, H. C. , & Denby, K. J. (2005). Secondary metabolites influence Arabidopsis/Botrytis interactions: Variation in host production and pathogen sensitivity. The Plant Journal, 44, 25–36. 10.1111/j.1365-313X.2005.02508.x 16167893

[pld3309-bib-0036] Konrad, S. S. , & Ott, T. (2015). Molecular principles of membrane microdomain targeting in plants. Trends in Plant Science, 20, 351–361. 10.1016/j.tplants.2015.03.016 25936559

[pld3309-bib-0037] Kumar, M. , Wightman, R. , Atanassov, I. , Gupta, A. , Hurst, C. H. , Hemsley, P. A. , & Turner, S. (2016). S‐Acylation of the cellulose synthase complex is essential for its plasma membrane localization. Science, 353, 166–169. 10.1126/science.aaf4009 27387950

[pld3309-bib-0038] Lenarčič, T. , Albert, I. , Böhm, H. , Hodnik, V. , Pirc, K. , Zavec, A. B. , Podobnik, M. , Pahovnik, D. , Žagar, E. , Pruitt, R. , Greimel, P. , Yamaji‐Hasegawa, A. , Kobayashi, T. , Zienkiewicz, A. , Gömann, J. , Mortimer, J. C. , Fang, L. , Mamode‐Cassim, A. , Deleu, M. , … Nürnberger, T. (2017). Eudicot plant‐specific sphingolipids determine host selectivity of microbial NLP cytolysins. Science, 358, 1431–1434. 10.1126/science.aan6874 29242345

[pld3309-bib-0039] Liang, Y. , Eudes, A. , Yogiswara, S. , Jing, B. , Benites, V. T. , Yamanaka, R. , Cheng‐Yue, C. , Baidoo, E. E. , Mortimer, J. C. , Scheller, H. V. , & Loqué, D. (2019). A screening method to identify efficient sgRNAs in Arabidopsis, used in conjunction with cell‐specific lignin reduction. Biotechnology for Biofuels, 12, 130. 10.1186/s13068-019-1467-y 31143243 PMC6532251

[pld3309-bib-0040] Liepman, A. H. , Wilkerson, C. G. , & Keegstra, K. (2005). Expression of cellulose synthase‐like (*Csl*) genes in insect cells reveals that *CslA* family members encode mannan synthases. Proceedings of the National Academy of Sciences of the United States of Anerica, 102, 2221–2226. 10.1073/pnas.0409179102 PMC548565 15647349

[pld3309-bib-0041] Luttgeharm, K. D. , Kimberlin, A. N. , Cahoon, R. E. , Cerny, R. L. , Napier, J. A. , Markham, J. E. , & Cahoon, E. B. (2015). Sphingolipid metabolism is strikingly different between pollen and leaf in Arabidopsis as revealed by compositional and gene expression profiling. Phytochemistry, 115, 121–129. 10.1016/j.phytochem.2015.02.019 25794895

[pld3309-bib-0042] Magnin‐Robert, M. , Le Bourse, D. , Markham, J. E. , Dorey, S. , Clément, C. , baillieul, F. , & Dhondt‐Cordelier, S. (2015). Modifications of sphingolipid content affect tolerance to hemibiotrophic and necrotrophic pathogens by modulating plant defense responses in arabidopsis. Plant Physiology, 169, 2255–2274. 10.1104/pp.15.01126 26378098 PMC4634087

[pld3309-bib-0043] Malinovsky, F. G. , Fangel, J. U. , & Willats, W. G. (2014). The role of the cell wall in plant immunity. Frontiers in Plant Science, 5, 178. 10.3389/fpls.2014.00178 24834069 PMC4018530

[pld3309-bib-0044] Markham, J. E. , & Jaworski, J. G. (2007). Rapid measurement of sphingolipids from *Arabidopsis thaliana* by reversed‐phase high‐performance liquid chromatography coupled to electrospray ionization tandem mass spectrometry. Rapid Communications in Mass Spectrometry, 21, 1304–1314. 10.1002/rcm.2962 17340572

[pld3309-bib-0045] Markham, J. E. , Li, J. , Cahoon, E. B. , & Jaworski, J. G. (2006). Separation and identification of major plant sphingolipid classes from leaves. Journal of Biological Chemistry, 281, 22684–22694. 10.1074/jbc.M604050200 16772288

[pld3309-bib-0046] Markham, J. E. , Lynch, D. V. , Napier, J. A. , Dunn, T. M. , & Cahoon, E. B. (2013). Plant sphingolipids: Function follows form. Current Opinion in Plant Biology. 10.1016/j.pbi.2013.02.009 23499054

[pld3309-bib-0047] Markham, J. E. , Molino, D. , Gissot, L. , Bellec, Y. , Hematy, K. , Marion, J. , Belcram, K. , Palauqui, J. C. , Satiat‐JeuneMaître, B. , & Faure, J. D. (2011). Sphingolipids containing very‐long‐chain Fatty acids define a secretory pathway for specific polar plasma membrane protein targeting in Arabidopsis. The Plant Cell, 23, 2362–2378. 21666002 10.1105/tpc.110.080473 PMC3160045

[pld3309-bib-0048] McFarlane, H. E. , Döring, A. , & Persson, S. (2014). The Cell Biology of Cellulose Synthesis. Annual Review of Plant Biology, 65, 69–94. 10.1146/annurev-arplant-050213-040240 24579997

[pld3309-bib-0049] Mortimer, J. C. , Miles, G. P. , Brown, D. M. , Zhang, Z. , Segura, M. P. , Weimar, T. , Yu, X. , Seffen, K. A. , Stephens, E. , Turner, S. R. , & Dupree, P. (2010). Absence of branches from xylan in Arabidopsis gux mutants reveals potential for simplification of lignocellulosic biomass. Proceedings of the National Academy of Sciences of the United States of America, 107, 17409–17414. 10.1073/pnas.1005456107 20852069 PMC2951434

[pld3309-bib-0050] Mortimer, J. C. , Yu, X. , Albrecht, S. , Sicilia, F. , Huichalaf, M. , Ampuero, D. , Michaelson, L. V. , Murphy, A. M. , Matsunaga, T. , Kurz, S. , & Stephens, E. (2013). Abnormal glycosphingolipid mannosylation triggers salicylic acid‐mediated responses in Arabidopsis. The Plant Cell, 25, 1881–1894. 23695979 10.1105/tpc.113.111500 PMC3694712

[pld3309-bib-0051] Nelson, B. K. , Cai, X. , & Nebenfuhr, A. (2007). A multicolored set of in vivo organelle markers for co‐localization studies in Arabidopsis and other plants. The Plant Journal, 51, 1126–1136. 17666025 10.1111/j.1365-313X.2007.03212.x

[pld3309-bib-0052] Niemann, M. C. , Bartrina, I. , Ashikov, A. , Weber, H. , Novak, O. , Spichal, L. , Strnad, M. , Strasser, R. , Bakker, H. , Schmülling, T. , & Werner, T. (2015). Arabidopsis ROCK1 transports UDP‐GlcNAc/UDP‐GalNAc and regulates ER protein quality control and cytokinin activity. Proceedings of the National Academy of Sciences of the United States of America, 112, 291–296. 25535363 10.1073/pnas.1419050112 PMC4291639

[pld3309-bib-0053] Norambuena, L. , Marchant, L. , Berninsone, P. , Hirschberg, C. B. , Silva, H. , & Orellana, A. (2002). Transport of UDP‐galactose in plants. Identification and functional characterization of AtUTr1, an Arabidopsis thaliana UDP‐galactos/UDP‐glucose transporter. Journal of Biological Chemistry, 277, 32923–32929. 10.1074/jbc.M204081200 12042319

[pld3309-bib-0054] Norambuena, L. , Nilo, R. , Handford, M. , Reyes, F. , Marchant, L. , Meisel, L. , & Orellana, A. (2005). AtUTr2 is an Arabidopsis thaliana nucleotide sugar transporter located in the Golgi apparatus capable of transporting UDP‐galactose. Planta, 222, 521–529. 10.1007/s00425-005-1557-x 15891899

[pld3309-bib-0055] Parker, J. L. , & Newstead, S. (2017). Structural basis of nucleotide sugar transport across the Golgi membrane. Nature, 551, 521–524. 10.1038/nature24464 29143814 PMC5701743

[pld3309-bib-0056] Parsons, H. T. , Christiansen, K. , Knierim, B. , Carroll, A. , Ito, J. , Batth, T. S. , Smith‐Moritz, A. M. , Morrison, S. , McInerney, P. , Hadi, M. Z. , Auer, M. , Mukhopadhyay, A. , Petzold, C. J. , Scheller, H. V. , Loqué, D. , & Heazlewood, J. L. (2012). Isolation and proteomic characterization of the Arabidopsis Golgi defines functional and novel components involved in plant cell wall biosynthesis. Plant Physiology, 159, 12–26. 10.1104/pp.111.193151 22430844 PMC3375956

[pld3309-bib-0057] Pau, G. , Fuchs, F. , Sklyar, O. , Boutros, M. , & Huber, W. (2010). EBImage–an R package for image processing with applications to cellular phenotypes. Bioinformatics, 26, 979–981. 10.1093/bioinformatics/btq046 20338898 PMC2844988

[pld3309-bib-0058] Plotnikova, J. M. , Reuber, T. L. , & Ausubel, F. M. (1998). Powdery mildew pathogenesis of Arabidopsis thaliana. Mycologia, 90, 1009–1016.

[pld3309-bib-0059] Prime, T. A. , Sherrier, D. J. , Mahon, P. , Packman, L. C. , & Dupree, P. (2000). A proteomic analysis of organelles from Arabidopsis thaliana. Electrophoresis, 21, 3488–3499. 10.1002/1522-2683(20001001)21:16<3488:AID-ELPS3488>3.0.CO;2-3 11079568

[pld3309-bib-0060] Ramirez, V. , Agorio, A. , Coego, A. , Garcia‐Andrade, J. , Hernandez, M. J. , Balaguer, B. , Ouwerkerk, P. B. , Zarra, I. , & Vera, P. (2011). MYB46 modulates disease susceptibility to Botrytis cinerea in Arabidopsis. Plant Physiology, 155, 1920–1935. 21282403 10.1104/pp.110.171843 PMC3091096

[pld3309-bib-0061] Rautengarten, C. , Birdseye, D. , Pattathil, S. , McFarlane, H. E. , Saez‐Aguayo, S. , Orellana, A. , Persson, S. , Hahn, M. G. , Scheller, H. V. , Heazlewood, J. L. , & Ebert, B. (2017). The elaborate route for UDP‐arabinose delivery into the Golgi of plants. Proceedings of the National Academy of Sciences of the United States of America, 114, 4261–4266. 10.1073/pnas.1701894114 28373556 PMC5402404

[pld3309-bib-0062] Rautengarten, C. , Ebert, B. , Liu, L. , Stonebloom, S. , Smith‐Moritz, A. M. , Pauly, M. , Orellana, A. , Scheller, H. V. , & Heazlewood, J. L. (2016). The Arabidopsis Golgi‐localized GDP‐L‐fucose transporter is required for plant development. Nature Communications, 7, 12119. 10.1038/ncomms12119 PMC4935801 27381418

[pld3309-bib-0063] Rautengarten, C. , Ebert, B. , Moreno, I. , Temple, H. , Herter, T. , Link, B. , Donas‐Cofre, D. , Moreno, A. , Saez‐Aguayo, S. , Blanco, F. , Mortimer, J. C. , Schultink, A. , Reiter, W.‐D. , Dupree, P. , Pauly, M. , Heazlewood, J. L. , Scheller, H. V. , & Orellana, A. (2014). The Golgi localized bifunctional UDP‐rhamnose/UDP‐galactose transporter family of Arabidopsis. Proceedings of the National Academy of Sciences of the United States of America, 111, 11563–11568. 10.1073/pnas.1406073111 25053812 PMC4128141

[pld3309-bib-0065] Rennie, E. A. , Ebert, B. , Miles, G. P. , Cahoon, R. E. , Christiansen, K. M. , Stonebloom, S. , Khatab, H. , Twell, D. , Petzold, C. J. , Adams, P. D. , & Dupree, P. (2014). Identification of a sphingolipid alpha‐glucuronosyltransferase that is essential for pollen function in Arabidopsis. The Plant Cell, 26, 3314–3325. 25122154 10.1105/tpc.114.129171 PMC4371831

[pld3309-bib-0066] Reyes, F. , Leon, G. , Donoso, M. , Brandizzi, F. , Weber, A. P. , & Orellana, A. (2010). The nucleotide sugar transporters AtUTr1 and AtUTr3 are required for the incorporation of UDP‐glucose into the endoplasmic reticulum, are essential for pollen development and are needed for embryo sac progress in Arabidopsis thaliana. The Plant Journal, 61, 423–435. 19906043 10.1111/j.1365-313X.2009.04066.x

[pld3309-bib-0067] Robert‐Seilaniantz, A. , Grant, M. , & Jones, J. D. (2011). Hormone crosstalk in plant disease and defense: More than just jasmonate‐salicylate antagonism. Annual Review of Phytopathology, 49, 317–343. 10.1146/annurev-phyto-073009-114447 21663438

[pld3309-bib-0068] Rollwitz, I. , Santaella, M. , Hille, D. , Flugge, U. I. , & Fischer, K. (2006). Characterization of AtNST‐KT1, a novel UDP‐galactose transporter from Arabidopsis thaliana. FEBS Letters, 580, 4246–4251. 16831428 10.1016/j.febslet.2006.06.082

[pld3309-bib-0069] Roudier, F. , Fernandez, A. G. , Fujita, M. , Himmelspach, R. , Borner, G. H. H. , Schindelman, G. , Song, S. , Baskin, T. I. , Dupree, P. , Wasteneys, G. O. , & Benfey, P. N. (2005). COBRA, an Arabidopsis extracellular glycosyl‐phosphatidyl inositol‐anchored protein, specifically controls highly anisotropic expansion through its involvement in cellulose microfibril orientation. The Plant Cell, 17, 1749–1763. 10.1105/tpc.105.031732 15849274 PMC1143074

[pld3309-bib-0070] Saez‐Aguayo, S. , Rautengarten, C. , Temple, H. , Sanhueza, D. , Ejsmentewicz, T. , Sandoval‐Ibañez, O. , Doñas, D. , Parra‐Rojas, J. P. , Ebert, B. , Lehner, A. , Mollet, J.‐C. , Dupree, P. , Scheller, H. V. , Heazlewood, J. L. , Reyes, F. C. , & Orellana, A. (2017). UUAT1 Is a golgi‐localized UDP‐uronic acid transporter that modulates the polysaccharide composition of arabidopsis seed mucilage. The Plant Cell, 29, 129–143. 10.1105/tpc.16.00465 28062750 PMC5304346

[pld3309-bib-0071] Schneider, C. A. , Rasband, W. S. , & Eliceiri, K. W. (2012). NIH Image to ImageJ: 25 years of image analysis. Nature Methods, 9, 671–675. 10.1038/nmeth.2089 22930834 PMC5554542

[pld3309-bib-0072] Sechet, J. , Htwe, S. , Urbanowicz, B. , Agyeman, A. , Feng, W. , Ishikawa, T. , Colomes, M. , Kumar, K. S. , Kawai‐Yamada, M. , Dinneny, J. R. , O'Neill, M. A. , & Mortimer, J. C. (2018). Suppression of Arabidopsis GGLT1 affects growth by reducing the L‐galactose content and borate cross‐linking of rhamnogalacturonan‐II. The Plant Journal, 96, 1036–1050. 10.1111/tpj.14088 30203879 PMC6263843

[pld3309-bib-0073] Skipski, V. P. (1975). Thin‐layer chromatography of neutral glycosphingolipids. Methods in Enzymology, 35, 396–425. 164604 10.1016/0076-6879(75)35178-1

[pld3309-bib-0074] Tellier, F. , Maia‐Grondard, A. , Schmitz‐Afonso, I. , & Faure, J. D. (2014). Comparative plant sphingolipidomic reveals specific lipids in seeds and oil. Phytochemistry, 103, 50–58. 10.1016/j.phytochem.2014.03.023 24731258

[pld3309-bib-0075] Wang, W. , Yang, X. , Tangchaiburana, S. , Ndeh, R. , Markham, J. E. , Tsegaye, Y. , Dunn, T. M. , Wang, G. L. , Bellizzi, M. , Parsons, J. F. , & Morrissey, D. (2008). An inositolphosphorylceramide synthase is involved in regulation of plant programmed cell death associated with defense in Arabidopsis. The Plant Cell, 20, 3163–3179. 19001565 10.1105/tpc.108.060053 PMC2613663

[pld3309-bib-0076] Wattelet‐Boyer, V. , Brocard, L. , Jonsson, K. , Esnay, N. , Joubès, J. , Domergue, F. , Mongrand, S. , Raikhel, N. , Bhalerao, R. P. , Moreau, P. , & Boutté, Y. (2016). Enrichment of hydroxylated C24‐and C26‐acyl‐chain sphingolipids mediates PIN2 apical sorting at trans‐Golgi network subdomains. Nature Communications, 7, 12788. 10.1038/ncomms12788 PMC5056404 27681606

[pld3309-bib-0077] Wightman, R. , & Turner, S. (2010). Trafficking of the plant cellulose synthase complex. Plant Physiology, 153, 427–432. 10.1104/pp.110.154666 20200066 PMC2879793

[pld3309-bib-0078] Wildermuth, M. C. , Dewdney, J. , Wu, G. , & Ausubel, F. M. (2001). Isochorismate synthase is required to synthesize salicylic acid for plant defence. Nature, 414, 562–565. 10.1038/35107108 11734859

[pld3309-bib-0079] Yan, D. , Yadav, S. R. , Paterlini, A. , Nicolas, W. J. , Petit, J. D. , Brocard, L. , Belevich, I. , Grison, M. S. , Vaten, A. , Karami, L. , el‐Showk, S. , Lee, J.‐Y. , Murawska, G. M. , Mortimer, J. , Knoblauch, M. , Jokitalo, E. , Markham, J. E. , Bayer, E. M. , & Helariutta, Y. (2019). Sphingolipid biosynthesis modulates plasmodesmal ultrastructure and phloem unloading. Nature Plants, 5, 604. 10.1038/s41477-019-0429-5 31182845 PMC6565433

