# Supplementary material for: The Arabidopsis thaliana nucleotide sugar transporter GONST2 is a functional homolog of GONST1
Source: Plant Direct. 2021 Mar 19;5(3):e00309. doi: 10.1002/pld3.309 (PMC7980081; doi:10.1002/pld3.309)
Supplement: Supplementary file 1 — FigS1 [file PLD3-5-e00309-s006.tif]

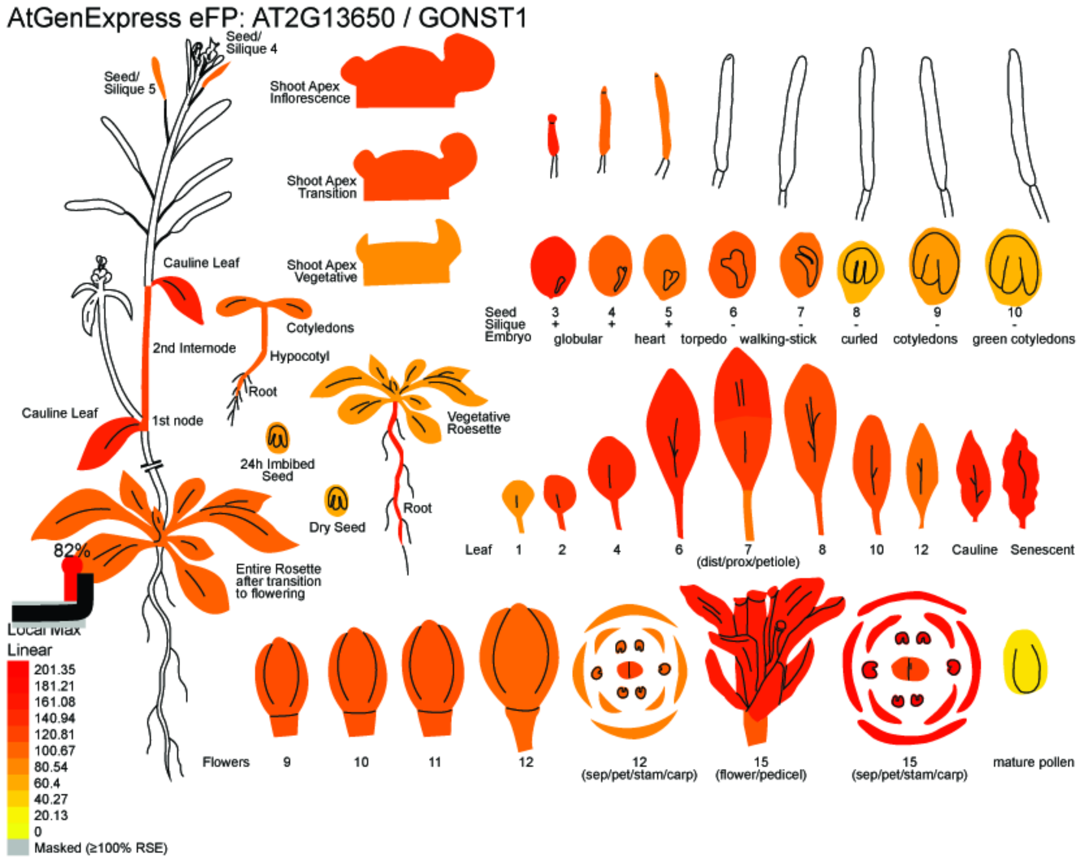

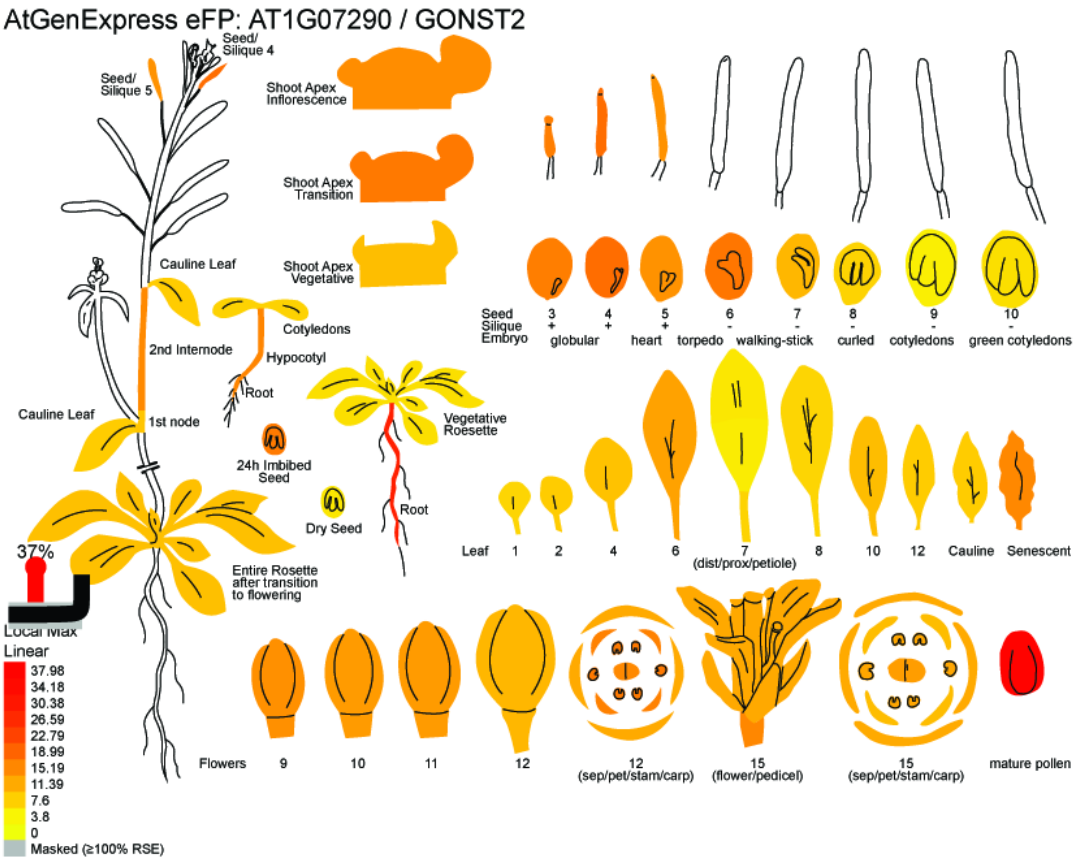

Supplemental Figure S1: Tissue-specific expression of *GONST2* and *GONST1*. Image was generated using the AtGenExpress eFP browser (Waese et al. 2017). Data are derived from Schmid et al. 2005 and Nakabayashi et al. 2005, and data were generated with the Affymetrix ATH1 array. Data are nomalized using GCOS, with a target intensity value (TGT) of 100.
