# Supplementary material for: The Arabidopsis thaliana nucleotide sugar transporter GONST2 is a functional homolog of GONST1
Source: Plant Direct. 2021 Mar 19;5(3):e00309. doi: 10.1002/pld3.309 (PMC7980081; doi:10.1002/pld3.309)
Supplement: Supplementary file 2 — FigS2 [file PLD3-5-e00309-s005.tif]

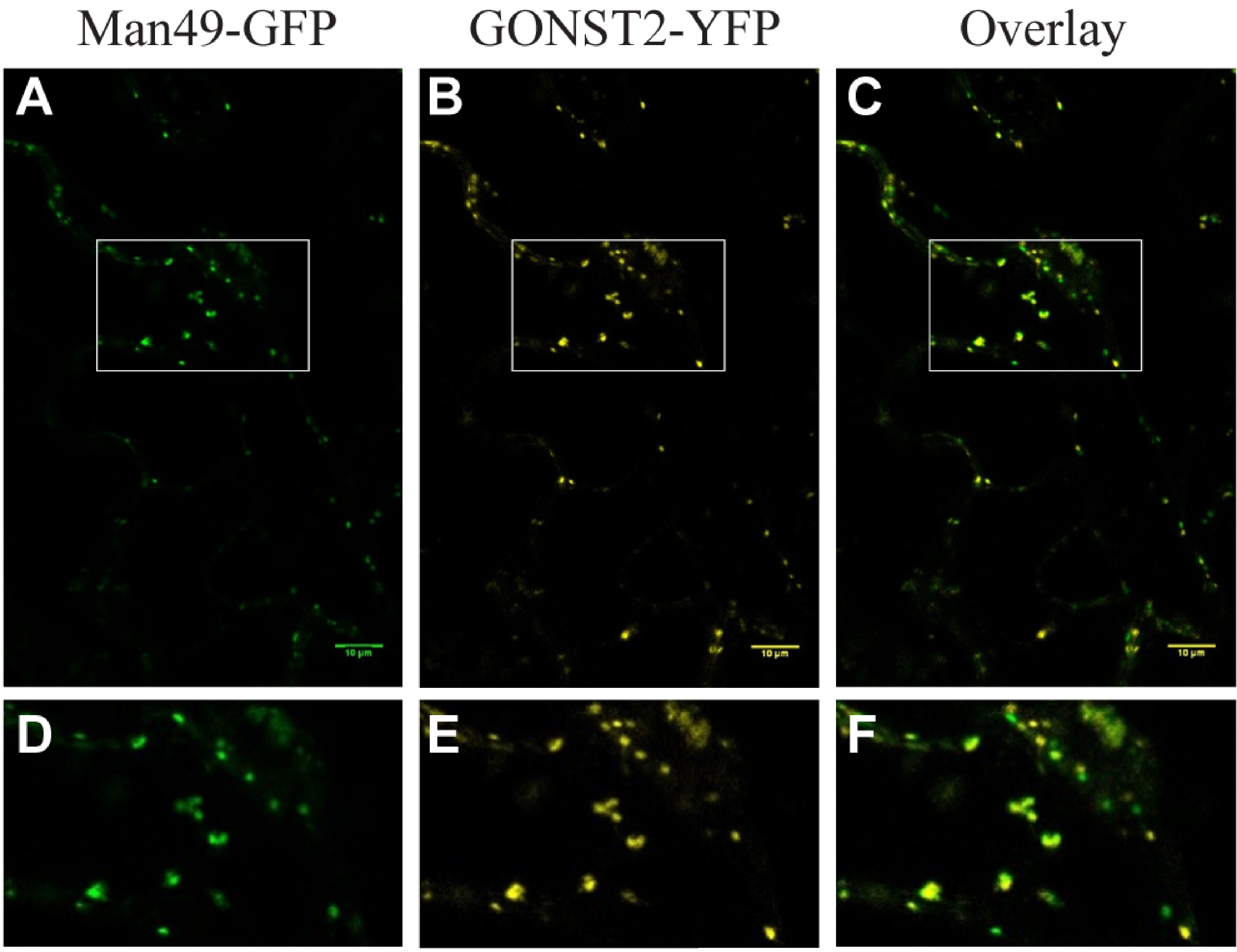

Supplemental Figure S2: Subcellular localization of GONST2. A *cis*-Golgi marker, Man49-GFP (A, D) along with a C-terminal YFP fusion of GONST2 (B, E), was co-infiltrated into tobacco leaves and expression of the fluorescent fusion proteins detected by confocal microscopy. An overlay of the two channels is shown in (C, F). D, E and F are 63X magnification of the white boxed area in A, B and C. Scale bar = 10 µm.
