# Supplementary material for: The Arabidopsis thaliana nucleotide sugar transporter GONST2 is a functional homolog of GONST1
Source: Plant Direct. 2021 Mar 19;5(3):e00309. doi: 10.1002/pld3.309 (PMC7980081; doi:10.1002/pld3.309)
Supplement: Supplementary file 3 — FigS3 [file PLD3-5-e00309-s007.tif]

A

GONST2_gRNA1 5'-GAGATAACAGGCGTGACCAC PAM GGG-3'

GONST2_gRNA2 3'-GTTTGGTGGGTTCATTAAACA PAM AGG-5'

B

gRNA1: gagataacaggcgtgac t cacggg

gRNA2: gtttggtgggttcattaa a acaagg

C

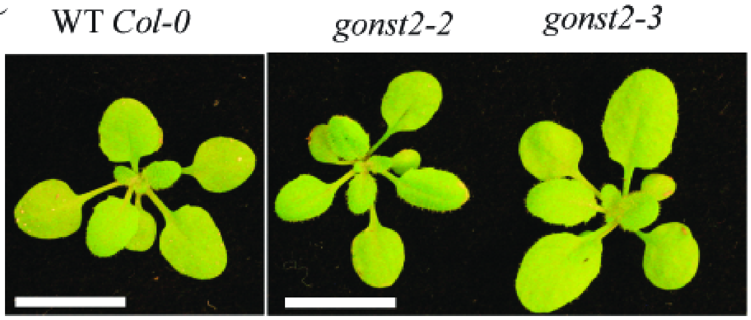

Supplemental Figure S3 Characterization of *gonst2-2* and *gonst2-3*. (A) gRNA sequences used to target *GONST2* (B) Sequence of *GONST2* gDNA for *gonst2-2* and *gonst2-3.* The genome edit is shown in red (C)15-day-old, agar grown WT-Col0, *gonst2-2* and *gonst2-3* seedlings. Scale bar = 1 cm.
