# Supplementary material for: The Arabidopsis thaliana nucleotide sugar transporter GONST2 is a functional homolog of GONST1
Source: Plant Direct. 2021 Mar 19;5(3):e00309. doi: 10.1002/pld3.309 (PMC7980081; doi:10.1002/pld3.309)
Supplement: Supplementary file 5 — FigS5 [file PLD3-5-e00309-s004.tif]

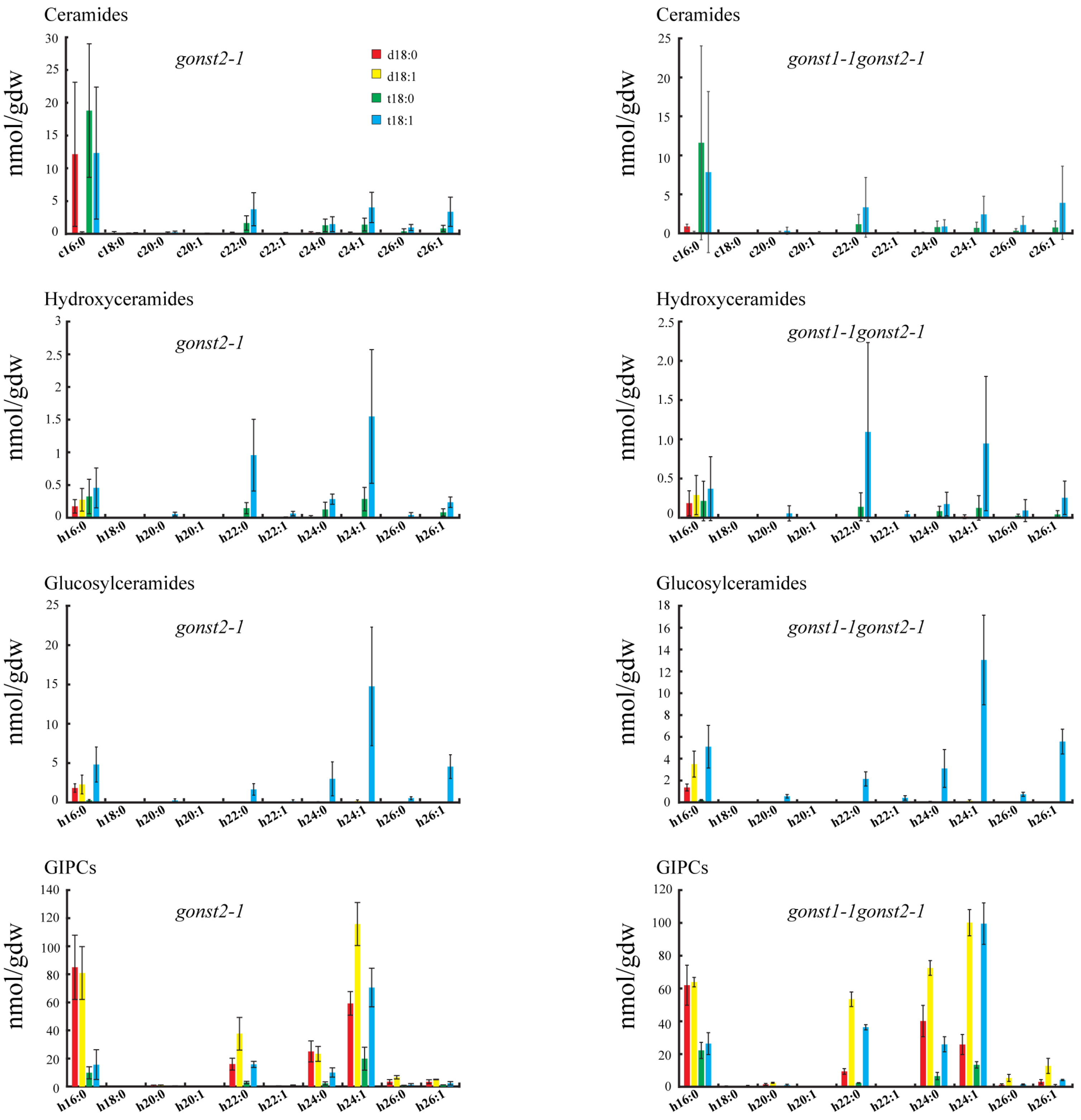

Supplemental Figure S5: Sphingolipidomic analysis of *gonst2-1* and *gonst1-1gonst2-1* callus. All analysis performed according to Fang *et al.* 2016. Data represents the average of 3 biological replicates, ±SD. See Supplemental Data Set 1 for the data used in preparation of this figure, as well for corresponding data for WT Ws and *gonst1-1*. The GIPC quantitation is an approximation, since the quantity of GlcA-IPCs and Hex_2-GlcA-IPCs were estimated using the response factors for Hex-GlcA-IPCs, as described in Supplemental Dataset 2.
