# Supplementary material for: The Arabidopsis thaliana nucleotide sugar transporter GONST2 is a functional homolog of GONST1
Source: Plant Direct. 2021 Mar 19;5(3):e00309. doi: 10.1002/pld3.309 (PMC7980081; doi:10.1002/pld3.309)
Supplement: Supplementary file 6 — FigS6 [file PLD3-5-e00309-s002.tif]

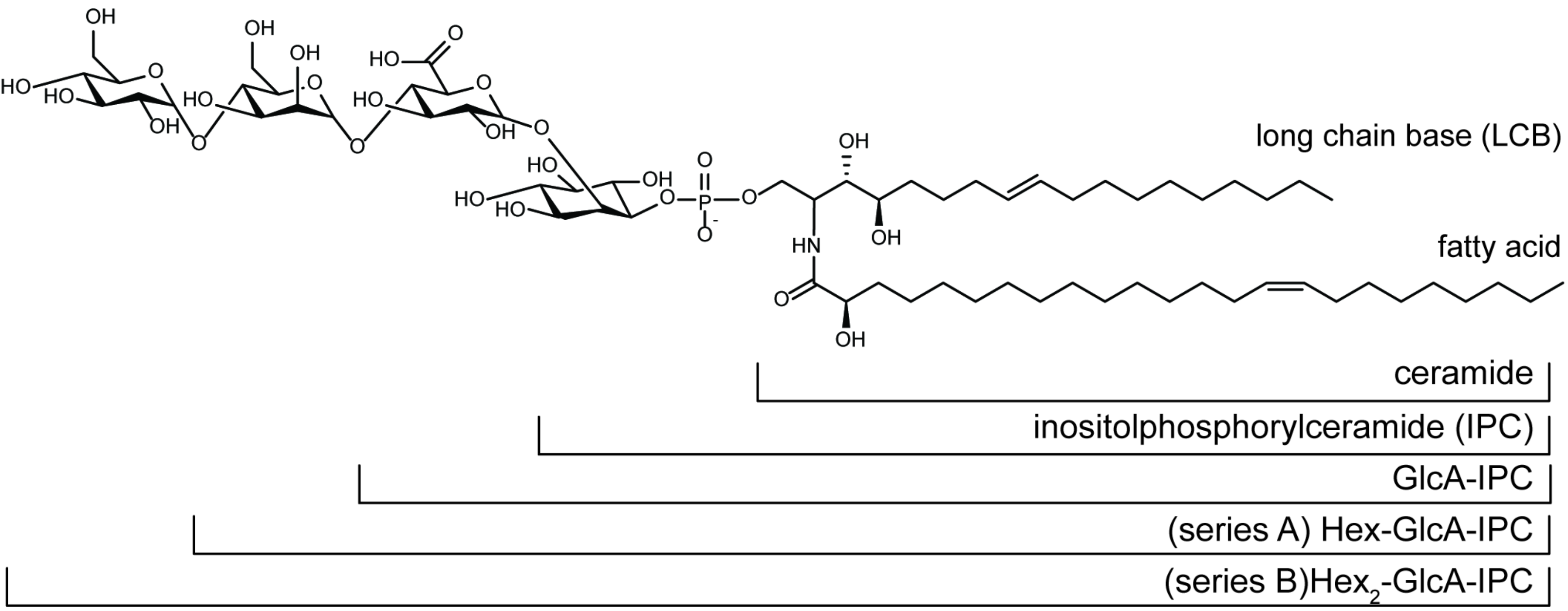

Supplemental Figure S6: Example GIPC structure, including the nomenclature used in this manuscript. Adapted from Fang *et al.* 2016. The ceramide t18:1/h24:1, is shown here, since it is the most abundant species in the Arabidopsis callus anaylzed in this study. The various sugar headgroup structures described in this article are also shown. It should be noted that although the final hexose is shown here as α-1,4-glucose, its identity is as yet unknown. The Series A/B nomenclature refers to that proposed by Mongrand and colleagues as describes e.g. in Mamode Cassim et al. 2019.
