# Supplementary material for: The Arabidopsis thaliana nucleotide sugar transporter GONST2 is a functional homolog of GONST1
Source: Plant Direct. 2021 Mar 19;5(3):e00309. doi: 10.1002/pld3.309 (PMC7980081; doi:10.1002/pld3.309)
Supplement: Supplementary file 7 — FigS7 [file PLD3-5-e00309-s009.tif]

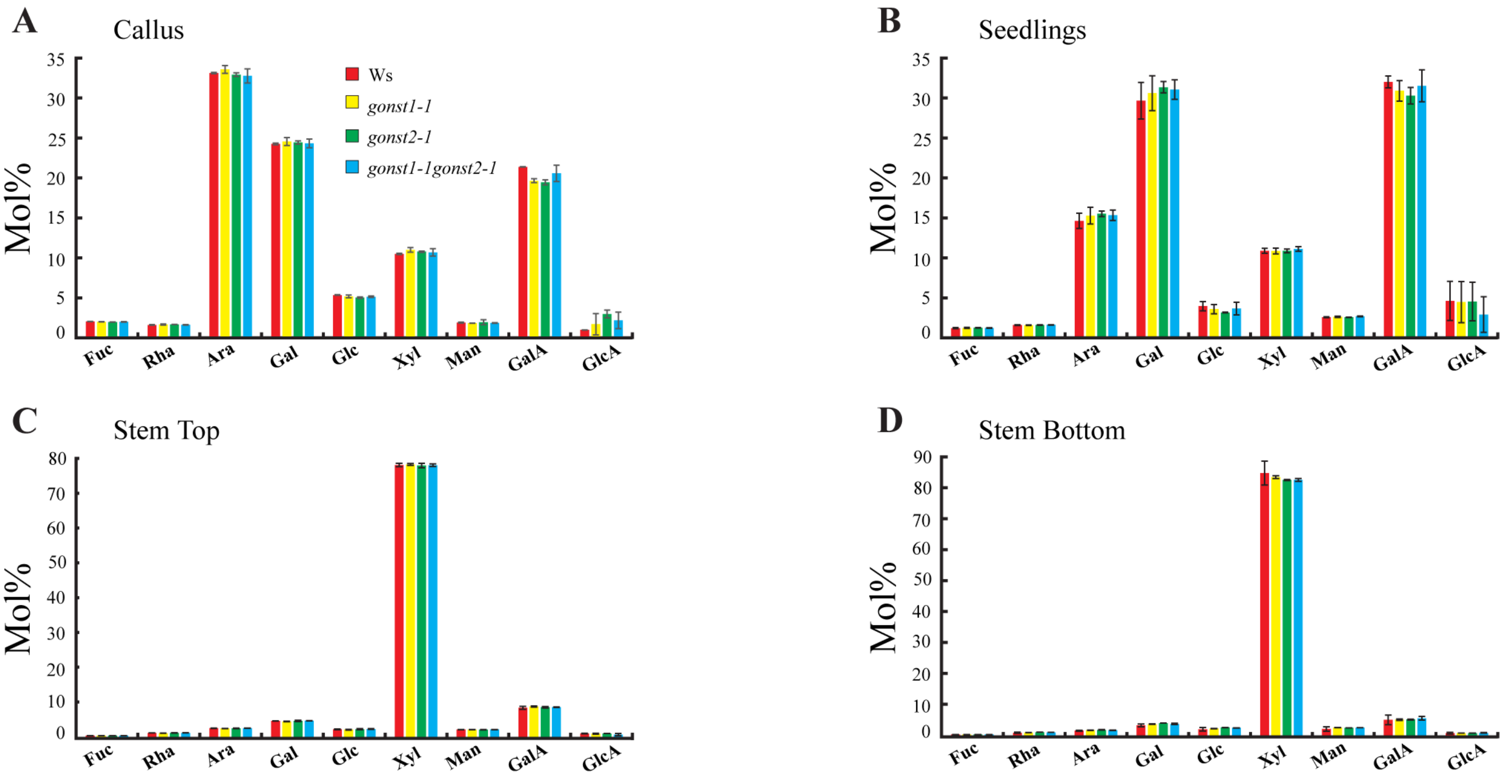

Supplemental Figure S7: Monosaccharide composition of non-cellulosic cell wall polysaccharides. AIR from 3 independently grown biological replicates was hydrolyzed with TFA and analyzed by HPAEC-PAD. Student's *t*-test was used to determine significant differences. All data is mean + SD of 3 independently grown biological replicates; asterisk indicate a significant difference between the two indicated genotypes. * $p < 0.05$, ** $p < 0.01$, *** $p < 0.001$. Data underlying these graphs are included in Supplemental Dataset S2.
