# Supplementary material for: The Arabidopsis thaliana nucleotide sugar transporter GONST2 is a functional homolog of GONST1
Source: Plant Direct. 2021 Mar 19;5(3):e00309. doi: 10.1002/pld3.309 (PMC7980081; doi:10.1002/pld3.309)
Supplement: Supplementary file 8 — TableS1 [file PLD3-5-e00309-s008.docx]

Supplemental Table S1: Oligonucleotide primers used in this project. All primers are shown 5'→3'.

**Primers used for Promoter swap experiment**	
GONST1 promoter	ccggaattcctctatgtcgattgatggtg
	ctagtctagatgcaaagctaagacctaccaaag
GONST2 Promoter	ccggaattcggagaccacctaaatcaatc
	ctagtctagattcttgttccagaatggcaac
GONST1 CDS	ctagtatagaatgaaattgtacgaacacgatgg
	acgcgtcgacggacttctccctcattttggc
GONST2 CDS	ctagtctagaatgtctgccgtgaaactggaag
	acgcgtcgactgacattttagctctggcaaag
**Primers used for Q-PCR**	
TUBULIN	acgtatcgatgtctatttcaacg
	atatcgtagagagcctcattgtcc
GONST2	gctttctgaccgcaagttattcac
	tcagcagcaccattgacacttc
**Primers used for generation of *gonst2-2* and *gonst2-3* using CRISPR/Cas9**	
GONST2_gRNA1_R (Pair with Piece1F)	TGTTTAATGAACCCACCAAACaatcgctatgtcgactctatc
Gonst2_gRNA1_F (Pair with Piece2R)	GTTTGGTGGGTTCATTAAACAgttttagagctagaaatagc
Sequencing of *gonst2-2*	tctttcaacttctcgatcag
	agatatctagtacagctaccac
Sequencing of *gonst2-3*	ggtagtggcaacagcaag
	tgacattttagctctggcaaag
