# Supplementary material for: The Arabidopsis thaliana nucleotide sugar transporter GONST2 is a functional homolog of GONST1
Source: Plant Direct. 2021 Mar 19;5(3):e00309. doi: 10.1002/pld3.309 (PMC7980081; doi:10.1002/pld3.309)
Supplement: Supplementary file 4 — FigS4 [file PLD3-5-e00309-s003.tif]

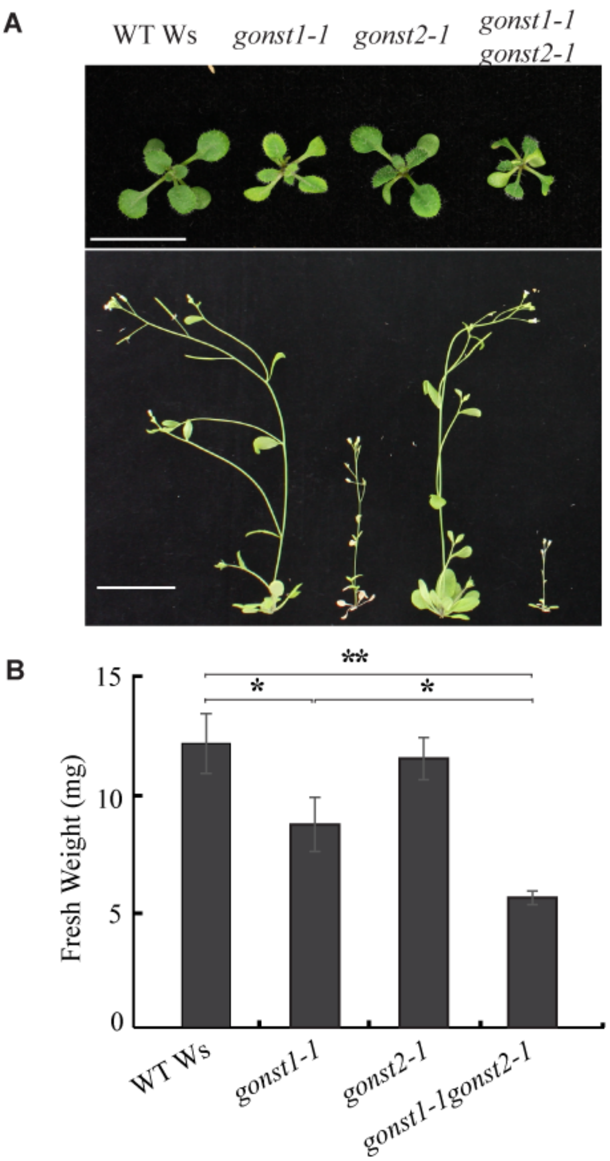

Supplemental Figure S4: Loss of GONST2 exacerbates the *gonst1* growth phenotype. (A) Top row: 15-day-old, agar grown WT, *gonst1-1*, *gonst2-1* and *gonst1-1gonst2-1* seedlings. Scale bar = 1 cm. Bottom row: 6-week-old WT, *gonst1-1*, *gonst2-1* and *gonst1-1gonst2-1.* Plants were first grown on agar for 10 days, and then transplanted onto soil. Bar = 3 cm. (B) Fresh weight of seedlings of 15-d-old, agar grown WT, *gonst1-1*, *gonst2-1* and *gonst1-1gonst2-1*. $n = 10$ individual plants grown simultaneously; ±SE; asterisk indicates significant difference from the wild type (Student's *t*-test, * $p < 0.05$, ** $p < 0.01$, *** $p < 0.001$).
